# Development of a hybrid model for a partially known intracellular signaling pathway through correction term estimation and neural network modeling

**DOI:** 10.1371/journal.pcbi.1008472

**Published:** 2020-12-14

**Authors:** Dongheon Lee, Arul Jayaraman, Joseph S. Kwon

**Affiliations:** 1 Artie McFerrin Department of Chemical Engineering, Texas A&M University, College Station, Texas, USA; 2 Texas A&M Energy Institute, Texas A&M University, College Station, Texas, USA; 3 Department of Biomedical Engineering, Texas A&M University, College Station, Texas, USA; University of Pennsylvania, UNITED STATES

## Abstract

Developing an accurate first-principle model is an important step in employing systems biology approaches to analyze an intracellular signaling pathway. However, an accurate first-principle model is difficult to be developed since it requires in-depth mechanistic understandings of the signaling pathway. Since underlying mechanisms such as the reaction network structure are not fully understood, significant discrepancy exists between predicted and actual signaling dynamics. Motivated by these considerations, this work proposes a hybrid modeling approach that combines a first-principle model and an artificial neural network (ANN) model so that predictions of the hybrid model surpass those of the original model. First, the proposed approach determines an optimal subset of model states whose dynamics should be corrected by the ANN by examining the correlation between each state and outputs through relative order. Second, an L2-regularized least-squares problem is solved to infer values of the correction terms that are necessary to minimize the discrepancy between the model predictions and available measurements. Third, an ANN is developed to generalize relationships between the values of the correction terms and the system dynamics. Lastly, the original first-principle model is coupled with the developed ANN to finalize the hybrid model development so that the model will possess generalized prediction capabilities while retaining the model interpretability. We have successfully validated the proposed methodology with two case studies, simplified apoptosis and lipopolysaccharide-induced NF*κ*B signaling pathways, to develop hybrid models with *in silico* and *in vitro* measurements, respectively.

## Author summary

An intracellular signaling pathway is often represented by a set of nonlinear ordinary differential equations, which translate our current knowledge about the signaling pathway into a testable mathematical model. However, predictions from such models are often subject to high uncertainty since many signaling pathways are only partially known beforehand. In this study, we propose a systematic approach to develop a hybrid model to improve model accuracy by combining machine learning and the first-principle modeling. Specifically, model correction terms are learned from discrepancy between model predictions and measurements, and these terms are added to the first-principle model to enhance the prediction accuracy. Once these correction terms are learned from the data, an artificial neural network (ANN) model is developed to find an empirical relation between the model and the correction terms so that the developed ANN can be used to posses improved predictive capabilities even in new operating conditions (i.e., generalizability). The final hybrid model is then constructed by coupling the first-principle model with the developed ANN.

## Introduction

An intracellular signaling pathway is a biochemical reaction network of cells to adjust their metabolism, gene expression, and other necessary actions so that the cells can appropriately respond to perturbations present in their environment [1, 2]. Since an intracellular signaling pathway is complex involving interactions among a large number of biomolecules inside a cell, it is common to implement a systems biology approach, which integrates experimental observations and mathematical modeling, to analyze the signaling pathway comprehensively [3, 4]. As a result, one of the key tasks in systems biology is to develop a predictive mathematical model for analyzing underlying mechanisms and generating new hypotheses to be tested in the future. To this end, a first-principle based mechanistic model has been a preferred choice for modeling an intracellular signaling pathway. Specifically, a system of nonlinear ordinary differential equations (ODEs) is constructed based on the current knowledge about a system, where its differential equations are derived using kinetic laws such as mass-action and Michaelis-Menten kinetics [4–6]. Since a first-principle model represents the current understandings of a system, its ODEs are physically meaningful, and its predictions are valid over a wide range of conditions [7, 8]. However, since a signaling pathway of interest is often only partially understood due to its inherent complexity, structural mismatches often exist between dynamics of the true system and those predicted by the corresponding first-principle model [4, 8–12].

Instead of a first-principle model, a data-driven model can be developed from available experimental measurements, which can describe the input-output dynamics adequately even when mechanistic understanding of the system is limited [13–17]. However, a data-driven model has narrow applicability as it is tailored to describe input-output relationships contained in the training datasets [18, 19]. Also, available measurements of an intracellular signaling pathway are often limited both in quantity and quality, which may further limit the direct application of a data-driven approach [20, 21].

As an alternative, a hybrid modeling approach that combines first-principle and data-driven modeling techniques has been proposed to describe a process that is only partially known [19]. Here, a hybrid model refers to an improved version of a first-principle model that compensates for model-system mismatches by introducing empirical parameters or terms, which are inferred from available measurements. As a result, a hybrid model has better prediction capabilities than a first-principle one while it preserves generalizability and interpretability, which are difficult to be achieved through a data-driven model [18, 22, 23]. A classic example of the hybrid modeling approach is to model a fedbatch bioreactor, where the biomass growth rate is estimated from process data and coupled with mass conversation laws [18, 22]. In these studies, the mass conservation laws represent our prior knowledge of the system (i.e., the first-principle model), whereas its growth rate is uncertain and inferred from the measurements to improve the model’s prediction accuracy. Due to its merits, the hybrid modeling approach has been implemented to model various biological and chemical processes such as bioprocess development and optimization [24, 25], modeling propagation of fractures during hydraulic fracturing process [7], transcription factor dynamics [8], thin film deposition [26], metallurgic processes [27, 28], and flour beetles population dynamics [29, 30].

In this work, we propose a systematic hybrid modeling approach for an intracellular signaling pathway by incorporating available mechanistic knowledge and experimental measurements. Compared to previous implementation of hybrid models, unique challenges arise when a hybrid model is developed for an intracellular signaling pathway. First, a first-principle model of an intracellular signaling pathway is usually high-dimensional with a large number of states while the amount of available measurements is usually limited. Hence, it is desirable to minimize the number of components in a hybrid model that should be inferred from experimental measurements to minimize the possibility of overfitting, which may compromise the generalizability of the hybrid model. At the same time, it is usually unknown beforehand which parts of a hybrid model should be represented by a data-driven model. In modeling a bioreactor, it is known that the largest uncertainty resides in the cell growth rate, and it is inferred from experimental measurements [18, 22]. However, such knowledge is usually not available for an intracellular signaling pathway [4, 21].

Motivated by the above considerations, we propose a systematic approach to construct a hybrid model to describe the dynamics of an intracellular signaling pathway, which is only partially known. Among a few possible hybrid model structures, a hybrid model formulation proposed by Engelhardt et al. [31, 32] is adopted, where differential equations of model states are adjusted by correction terms inferred from experiments. Since an intracellular signaling pathway is often high-dimensional and its origin of prediction inaccuracy is unknown beforehand, a graphical approach is implemented to determine a subset of model states that have the highest correlations with the measurements. And only these states’ dynamics are modified by the correction terms. Specifically, the values of the correction terms are estimated at the times when the measurements are available so that the model with the estimated correction terms can reproduce the measurements accurately. Then, an empirical map between the first-principle model and the correction terms is approximated by an artificial neural network (ANN). Once an ANN is trained, the hybrid model now can be constructed by integrating the first-principle model and the ANN. The effectiveness and feasibility of the proposed methodology are demonstrated by developing hybrid models of intracellular signaling pathways for two case studies.

## Methods

### System description

Dynamics of an intracellular signaling pathway are described by a system of nonlinear ODEs as follows:

$$\begin{array}{c} \begin{array}{cc} \dot{x} & =f\left(x,\theta ,u;t\right),\;x\left(0\right)=x_{0}\\ y & =g(x,u;t) \end{array} \end{array}$$ (1)

where $x\in R^{n_{x}}$ is the state vector, $\theta \in R^{n_{\theta }}$ is the parameter vector, ***x***_0_ is the initial value of the state vector ***x***, and $y\in R^{n_{y}}$ is the output vector.

Such ODE models for an intracellular signaling pathway are formulated based on the current understandings of the underlying signaling pathway. Hence, the accuracy of an ODE model depends on the accuracy and completeness of the prior knowledge. Unfortunately, an intracellular signaling pathway is quite complex, which involves interactions among a large number of intracellular biomolecules. As a result, it is likely to have a model-system mismatch, which prevents from utilizing the model for system analysis and prediction.

Under this circumstance, the following hybrid model can be used to minimize potential model-system mismatches while preserving the available knowledge of the system [31, 32]:

$$\begin{array}{c} \begin{array}{cc} \dot{\tilde{x}} & =f\left(\tilde{x},\theta ,u;t\right)+w\left(t\right),\;\tilde{x}\left(0\right)=x_{0}\\ \tilde{y} & =g(\tilde{x},u;t) \end{array} \end{array}$$ (2)

where $\tilde{x}\in R^{n_{x}}$ and $\tilde{y}\in R^{n_{y}}$ are the state and output vectors of the hybrid model, respectively, *u* is the external input, and $w\left(t\right)\in R^{n_{x}}$ is the vector of correction terms introduced to improve the overall model prediction accuracy.

In order for Eq 2 to properly predict true dynamics of the system, the values of ***w*** need to be explicitly known at any arbitrary time instants so that the hybrid model (Eq 2) can be numerically integrated. Therefore, ***w*** values are inferred from available experimental measurements. On the other hand, even with experimentally inferred ***w***(*t*), the hybrid model cannot be used to predict the system dynamics under a new condition since the corresponding temporal profile of ***w*** are not available. Hence, this study aims to develop a functional map H that can compute the value of ***w*** at time *t* for given values of the model states ***x***(*t*) and *t*; that is, we aim to develop w(t)=H(x(t);t) for prediction generalizability of the hybrid model.

In summary, this study aims to develop a hybrid model by the following two subsequent steps:

1. Infer ***w***(*t*) from available experimental measurements.
2. Develop the function, H, that maps from ***x*** and *t* to ***w***(*t*).

### Estimation of *w*(*t*)

Suppose that measurements are obtained at *N*_*t*_ discrete time instants (i.e., $t_{s}=\left[0,\ldots ,t_{N_{t}}\right]$) under *N*_*u*_ different *u*. Then, the estimation of ***w***(***t***) can be formulated into the following minimization problem:

$$\underset{w^{1}\left(t\right),\ldots ,w^{N_{u}}\left(t\right)}{\text{min}}\;\;\sum_{s=1}^{N_{u}}\sum_{l=1}^{N_{t}}\sum_{i=1}^{n_{y}}{\left(\frac{{\tilde{y}}_{i}\left(u_{s};t_{l}\right)-{\hat{y}}_{i}\left(u_{s};t_{l}\right)}{{\hat{y}}_{i}\left(u_{s};t_{l}\right)}\right)}^{2}$$ (3a)

$$\begin{array}{cc} s.t.\; & \dot{\tilde{x}}=f(\tilde{x},u_{s};t)+w^{s}\left(t\right)\;\tilde{x}\left(0\right)=x_{0} \end{array}$$ (3b)

$$\begin{array}{cc} & \tilde{y}\left(t_{l}\right)=g(\tilde{x}\left(t_{l}\right),u_{s};t_{l}) \end{array}$$ (3c)

where ***w***^*s*^(***t***) is the continuous temporal profile of ***w***(*t*) from *t* = 0 to $t_{N_{t}}$ under input *u*_*s*_, and ${\hat{y}}_{i}\left(u_{s};t\right)$ is the *i*^th^ output measured under input *u*_*s*_ at time *t*.

However, Eq 3 is likely to be ill-conditioned because (1) it is an infinite dimensional problem in which the decision variables (i.e., ***w***(*t*)) are continuous temporal profiles, and (2) the available measurements are limited in quantity (i.e., small values of *n*_*y*_ and *N*_*t*_) [33–36]. Hence, its solution is subject to high uncertainties, and the resulted hybrid model based on the estimated ***w***(***t***) will be difficult to be generalized for future predictions.

#### Proposed methodology for estimating *w* from measurements

In order to address the aforementioned ill-posedness, the following assumptions are made to reduce the dimension of the estimation problem. First, a L2-regularized least-squares problem is solved to estimate ***w*** by handling potential overfitting issues [37–39]. Second, this study aims to infer the values of ***w*** at the time instants only when the measurements are available (i.e., ***t***_*s*_). Third, instead of adding correction terms to all the model states, correction terms are given to only a subset of states, which will be denoted as $x_{c}\in R^{n_{s}}$ *n*_*s*_ < *n*_*x*_, is selected a priori. Lastly, a linear interpolation is used to compute values of the correction terms at time *t*, when the measurements are not available (i.e., *t* ∉ ***t***_*s*_), as follows:

$$\begin{array}{c} w_{c,i}\left(t\right)=w_{c,i}\left(t_{l}\right)+\left(t-t_{l}\right)\frac{w_{c,i}\left(t_{l+1}\right)-w_{c,i}\left(t_{l}\right)}{t_{l+1}-t_{l}},\;t_{l}<t<t_{l+1},\;\forall i=1,\ldots ,n_{x} \end{array}$$ (4)

where *w*_*c*,*i*_ is the *i*^th^ correction term, *t*_*l*_ is the latest time point of ***t***_*s*_ preceding *t*, and *t*_*l*+1_ is the earliest time point in ***t***_*s*_ following *t*.

With these assumptions, the estimation of ***w***(***t***) is reformulated as follows:

$$\underset{W}{\text{min}}\;\sum_{s=1}^{N_{u}}\sum_{l=1}^{N_{t}}\sum_{i=1}^{n_{y}}{\left(\frac{{\tilde{y}}_{i}(u_{s};t_{l})-{\hat{y}}_{i}(u_{s};t_{l})}{{\hat{y}}_{i}(u_{s};t_{l})}\right)}^{2}+R(W)$$ (5a)

$$\text{s}.\text{t}.\;\dot{\tilde{x}}=f(\tilde{x},\theta ,u_{s};t)+H[\begin{array}{c} w_{c,1}^{s}(t)\\ \vdots \\ w_{c,n_{s}}^{s}(t) \end{array}],\;\tilde{x}(0)=x_{0}$$ (5b)

$$\tilde{y}\left(t_{l}\right)=g(\tilde{x}\left(t_{l}\right),u_{s};t_{l})$$ (5c)

$$R\left(W\right)=\frac{\alpha }{2}\sum_{s=1}^{N_{u}}\sum_{l=1}^{N_{t}}{∥w_{c}^{s}\left(t_{l}\right)∥}^{2}$$ (5d)

$$W=[w_{c,1}^{1}(t_{1})\;\cdots \;w_{c,1}^{1}(t_{N_{t}})\;w_{c,1}^{2}(t_{1})\;\cdots \;w_{c,1}^{N_{u}}(t_{1})\;\cdots \;w_{c,n_{s}}^{N_{u}}(t_{N_{t}})]$$ (5e)

$$H_{ij}=\{\begin{array}{ll} 1 & \text{if}\;x_{i}=x_{c_{j}},\;i=1,\ldots ,n_{x},\;j=1,\ldots ,n_{s}\\ 0 & \text{otherwise}. \end{array}$$ (5f)

where ***H*** is a *n*_*x*_ × *n*_*s*_ matrix, $x_{c}={\left[x_{c,1},\cdots ,x_{c,n_{s}}\right]}^{T}\in R^{n_{s}}$ is a subset of ***x*** whose dynamics are corrected by $w_{c}={\left[w_{c,1},\cdots ,w_{c,n_{s}}\right]}^{T}\in R^{n_{s}}$, *H*_*ij*_ is the entry in ***H*** at the *i*^th^ row and the *j*^th^ column, and *α* is the L2-regularization tuning parameter. It should be noted that the above assumptions are introduced to estimate the dynamics of *w*(*t*) minimizing the likelihood of the overfitting by interpolating the values of *w*(*t*) at the time instants when the measurements are not available. Hence, the overall accuracy of the inferred *w*(*t*) is likely to improve if the outputs are measured more frequently, which in turn increases the overall prediction power. Also, Eqs 5b and 5c assume that each correction term is added to only one state’s differential equation. As mentioned before, values of ***w***_*c*_ are linearly interpolated to solve Eqs 5b and 5c at time instants when measurements are not available.

In Eq 5, the optimal value of *α* is unknown beforehand, so its value is optimized by five-fold cross-validation. Specifically, the available measurements $Y=\hat{y}$ are divided into training and validation datasets with a 8:2 ratio in five different ways, and the regularized least-square problem (Eq 5) is solved with one particular value of *α* with respect to each of the five training datasets. Then, the optimal value of *α* is chosen by examining average model errors with respect to both the training and validation datasets.

#### Selection of *x*_*c*_

A key step before solving Eq 5 is to identify ***x***_*c*_, whose trajectories are corrected by ***w***_*c*_. Specifically, two questions need to be addressed: first, what is the dimension of ***x***_*c*_ (i.e., *n*_*s*_), and second, when the value of *n*_*s*_ is known, which states in ***x*** should be selected to form ***x***_*c*_. In this study, we employ the idea of invertibility and a graph-theoretical approach to determine ***x***_*c*_.

Specifically, we choose ***x***_*c*_ from ***x*** so that the resulted system is close to be invertible [40]. If a system is invertible, for a given value of ***x***_0_, unique values of ***y*** will correspond to unique values of inputs, so one could reconstruct the values of inputs from available output measurements [41, 42]. If ***w***_*c*_ in the hybrid model (Eqs 5b and 5c) is viewed as an input to the system and the hybrid model is invertible, the values of ***w***_*c*_ can be uniquely characterized from given measurements [41, 43]. Hence, it is our best interest to select the dimension of ***w***_*c*_ as well as its placement so that the resulted hybrid model is invertible, which will attenuate the ill-posedness of the inverse problem (i.e., Eq 5). In this regard, Daoutidis and Kravaris [41] have shown that a dynamic system is invertible when the following matrix is nonsingular:

$$\begin{array}{c} C\left(x\right)=[\begin{array}{ccc} L_{h_{1}}L_{f}^{r_{1}-1}g_{1}\left(x\right) & \cdots & L_{h_{n_{s}}}L_{f}^{r_{1}-1}g_{1}\left(x\right)\\ \vdots & \ddots & \vdots \\ L_{h_{1}}L_{f}^{r_{n_{y}}-1}g_{n_{y}}\left(x\right) & \cdots & L_{h_{n_{s}}}L_{f}^{r_{n_{y}}-1}g_{n_{y}}\left(x\right) \end{array}]=[\begin{array}{ccc} c_{11} & \cdots & c_{1n_{s}}\\ \vdots & \ddots & \vdots \\ c_{n_{y}1} & \cdots & c_{n_{y}n_{s}} \end{array}] \end{array}$$ (6)

where ***C***(*x*) is the characteristic matrix of the system [44], L represents Lie derivative defined as $L_{f}g_{i}\left(x\right)=\sum_{j=1}^{n_{x}}\left(\partial g_{i}/\partial x_{j}\right)f_{j}\left(x\right)$, *h*_*k*_ is the *k*^th^ column vector of the matrix ***H*** in Eqs 5b and 5c, where *k* = 1, …, *n*_*s*_, and *r*_*i*_ is the relative order of output *y*_*i*_ with respect to ***w***_*c*_, which is defined as the smallest integer for which

$$\begin{array}{c} \left[\begin{array}{ccc} L_{h_{1}}L_{f}^{r_{i}-1}g_{i}\left(x\right) & \cdots & L_{h_{n_{s}}}L_{f}^{r_{i}-1}g_{i}\left(x\right) \end{array}]\neq [\begin{array}{ccc} 0 & \cdots & 0 \end{array}\right] \end{array}$$ (7)

or *r*_*i*_ = ∞, if such integer does not exist [43]. Additionally, the following relation holds true for *r*_*i*_:

$$\begin{array}{c} r_{i}=\text{min}\left(r_{i1},r_{i2},\cdots ,r_{in_{s}}\right) \end{array}$$ (8)

where *r*_*ij*_ is the relative order of *y*_*i*_ with respect to *w*_*c*,*j*_, which is the smallest integer for which $L_{h_{j}}L_{f}^{r_{ij}-1}g_{i}\left(x\right)\neq 0$ or *r*_*ij*_ = ∞ if such integer does not exist. Based on the definition of the relative order, the relative order matrix of Eqs 5b and 5c can be defined as follows:

$$\begin{array}{c} R=[\begin{array}{cccc} r_{11} & r_{12} & \cdots & r_{1n_{s}}\\ r_{21} & r_{22} & \cdots & r_{2n_{s}}\\ \vdots & \cdots & \ddots & \vdots \\ r_{n_{y}1} & r_{n_{y}2} & \cdots & r_{n_{y}n_{s}} \end{array}] \end{array}$$ (9)

In this study, ***x***_*c*_ is chosen so that *C*(***x***) of Eqs 5b and 5c is nonsingular, which will minimize the ill-posedness of the inverse problem (Eq 5). First, we let the size of ***x***_*c*_ equal to the size of outputs (i.e., *n*_*s*_ = *n*_*y*_) so that *C*(***x***) is square. Second, an optimal choice of ***x***_*c*_ is identified systematically from ***x***. In this regard, this study will assess the optimality of ***x***_*c*_ through the following criteria that are based on the relative order:

1. The value of $\sum_{i=1}^{n_{s}}r_{i}$ is minimum.
2. The value of $\sum_{i}^{n_{s}}\sum_{j\neq i}r_{i}/r_{ij}$ is minimum.

Previous studies have demonstrated that the relative order measures ‘physical closeness’ between a correction term and an output [43, 45]: a smaller value of *r*_*i*_ represents a stronger connection between ***w***_*c*_ and *y*_*i*_. So, the first criterion renders ***w***_*c*_ to have the maximum correlations with the outputs. On the other hand, the second criterion is to render each *w*_*c*,*j*_ will have the strongest correlation with only one output while having the weakest correlation with the remaining outputs [45, 46]. Consequently, by meeting the above two criteria, each correction term in ***w***_*c*_ will have the strongest correlation with only one correction term, which will minimize the likelihood of the ill-posedness of Eq 5 [47].

In the perspective of the invertibility, selecting ***x***_*c*_ based on the above two criteria, particularly the second one, maximizes the likelihood of *C*(***x***) to be nonsingular. To understand this point more clearly, the outputs and ***x***_*c*_ are re-ordered so that the smallest element in each row is diagonally located in the relative order matrix, and *C*(***x***) is also rearranged correspondingly. By minimizing the second criterion, the possibility that values of all *r*_*ij*_, ∀*j* ≠ *i*, are larger than *r*_*i*_ is maximized; therefore, the non-diagonal entries in the rearranged *C*(***x***) are likely to be zero. As a result, *C*(***x***) will be close to be a square diagonal matrix. Thus, achieving the aforementioned two criteria would guarantee the hybrid model (Eqs 5b and 5c) to be invertible as well as the reliability of the solution to Eq 5.

In order to select a combination based on the above criteria, the following steps are taken:

1. Enumerate all *n*_*y*_ permutations of ***x***.
2. For each candidate, construct the corresponding relative order matrix, and compute its $\sum_{i=1}^{n_{y}}r_{i}$.
3. Compute their ∑_*i*_ ∑_*j*≠*i*_ *r*_*i*_/*r*_*ij*_ values.
4. Find the candidate that satisfies the condition.

For implementing the above procedure, a relative order matrix has to be constructed for each candidate to calculate the two criteria. However, performing Lie differentiation can be computationally expensive; hence, a graphical approach is implemented to evaluate relative orders of a system [43], which will be discussed in the next section.

#### A graphical approach to evaluate relative orders

A state-space model of a process (Eqs 5b and 5c) can be represented by a directed graph, which is defined by a set of vertices and a set of edges by the following rules [43, 48]:

- States ($x\in R^{n_{x}}$), outputs ($y\in R^{n_{y}}$), and manipulated inputs ($w_{c}\in R^{n_{s}}$) are represented by a set of vertices in a graph.
- If ∂*f*_*i*_(***x***)/∂*x*_*j*_ ≠ 0, *i*, *j* = 1, …, *n*_*x*_, there is a unidirectional edge pointing from the vertex of *x*_*j*_ to that of *x*_*i*_.
- If ∂*f*_*i*_(***x***)/∂*w*_*c*,*k*_ ≠ 0, *k* = 1, …, *n*_*s*_, there is a unidirectional edge pointing from the vertex of *w*_*c*,*k*_ to that of *x*_*i*_.
- If ∂*y*_*l*_/∂*x*_*j*_ ≠ 0, *l* = 1, …, *n*_*y*_, there is a unidirectional edge pointing from the vertex of *x*_*j*_ to that of *y*_*l*_.
- A path from one vertex to another is a sequence of edges without repeating vertices, and the path length is the number of edges included in one particular path.

**Fig 1:**
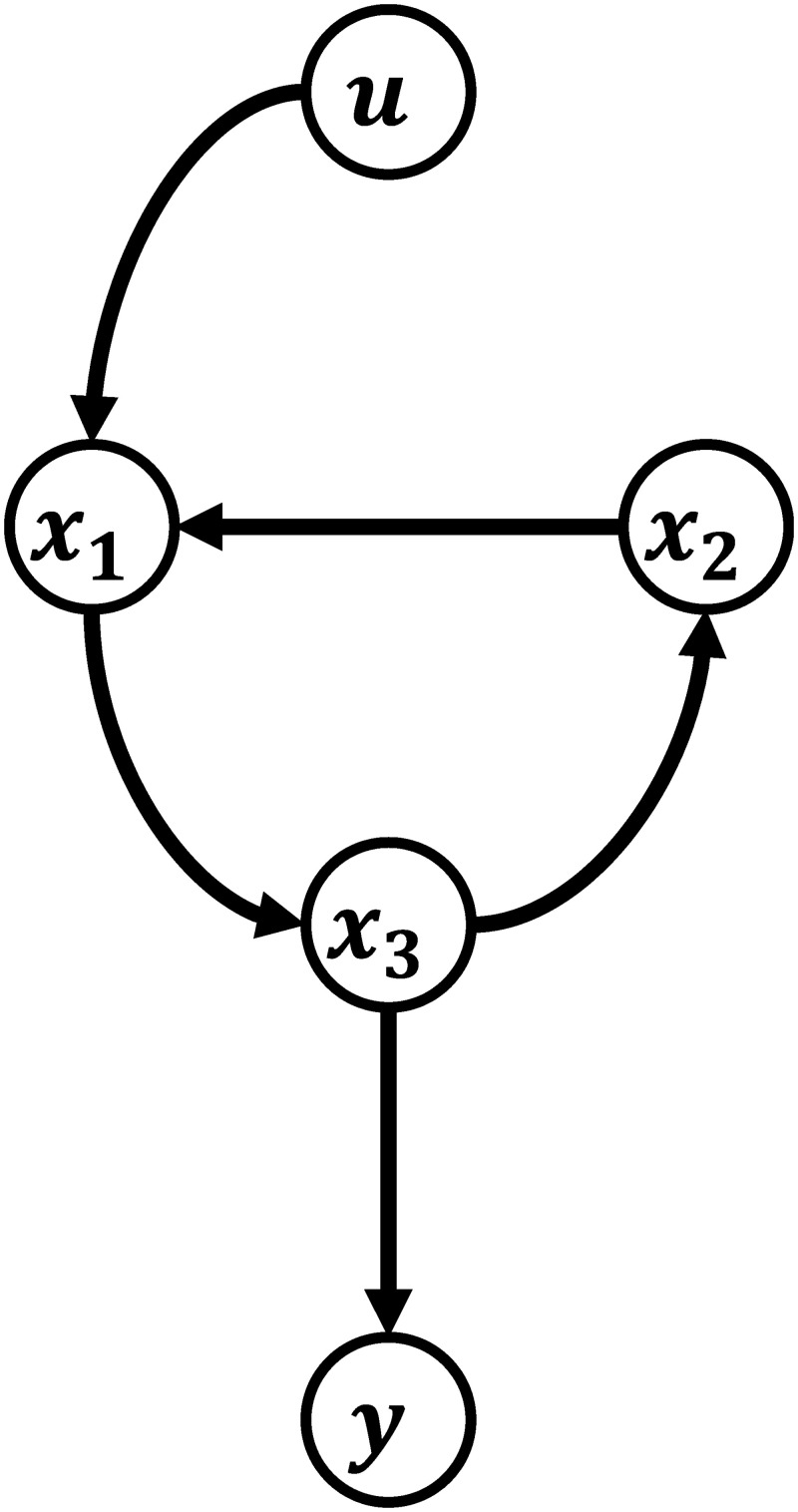
Schematic illustration of the directional graph of a state-space model.

Previously, Daoutidis and Kravaris [43] demonstrated that *r*_*ij*_ can be calculated by computing the shortest path length from an input *w*_*c*,*j*_ to an output *y*_*i*_ as follows:

$$\begin{array}{c} r_{ij}=l_{ij}-1 \end{array}$$ (10)

where *l*_*ij*_ is the shortest path length from an input *w*_*c*,*j*_ to an output *y*_*i*_. Therefore, the relative order matrix can be easily computed once a graph of a state-space model is constructed. As an example, Fig 1 provides an example on how a state-space model is translated into its corresponding directional graph; specifically, Fig 1 is a representation of the following dynamic system:

$$\begin{array}{c} \begin{array}{cc} \frac{dx_{1}}{dt} & =f_{1}\left(x_{2}\right)+u\\ \frac{dx_{2}}{dt} & =f_{2}\left(x_{3}\right)\\ \frac{dx_{3}}{dt} & =f_{3}\left(x_{1}\right)\\ y & =g(x_{3}) \end{array} \end{array}$$ (11)

The relative order of this system is two, which can be easily computed from its graph.

In summary, the procedures for selecting ***x***_*c*_, whose dynamics will be corrected by ***w***_*c*_, are as follows:

1. Set *n*_*s*_, the size of ***x***_*c*_, to be equal to the dimension of outputs (i.e., *n*_*y*_).
2. Enumerate all *n*_*s*_ permutations of ***x*** as candidates for ***x***_*c*_.
3. Construct a directional graph by adding correction terms to each ***x***_*c*_ candidate enumerated in the previous step.
4. Construct the corresponding relative order matrix based on Eq 10.
5. Find a configuration that has the lowest $\sum_{i=1}^{n_{y}}r_{i}$ and ∑_*i*_ ∑_*j*≠*i*_ *r*_*i*_/*r*_*ij*_ values.

Once the optimal ***x***_*c*_ is chosen, Eq 5 is solved to infer ***W***.

It should be noted that the identification of ***x***_*c*_ by the relative order and graph theory can also guide the future model refinement. Specifically, since the identified ***x***_*c*_ has the highest correlations with the output, further literature survey and experimentation on these states can be implemented to improve the differential equations for these states and thus increase the overall prediction accuracy of the first-principle model.

### Development of artificial neural network models

Once ***W*** is estimated by solving Eq 5, the available (imperfect) first-principle model coupled with the estimated ***W*** is now able to predict the system dynamics under the experimental measurements more accurately. However, it cannot predict system dynamics under a new operating condition since its corresponding ***w***_*c*_(***t***) is not available. Hence, the next step is to infer H, which maps ***x*** and current time *t*, to ***w***_*c*_(***t***), to generalize the model prediction so that the resulted hybrid model can predict the system dynamics under new conditions. In this regard, multiple ***w***_*c*_(***t***) should be obtained under a few different operating conditions (i.e., *N*_*u*_ > 1) so that the empirical function H mapping ***x*** and *t* to ***w***_*c*_(*t*) is accurate.

However, the functional form of H is usually unknown a priori. Although there are some methods proposed in the literature to identify functional forms from the data, inferring functional forms usually requires a large amount of data and can be computationally expensive [49–54]. Instead, we assume H to be an ANN model. Here, an ANN is chosen here due to its proven ability to represent any arbitrary input-output relations with sufficient accuracy [55, 56].

**Fig 2:**
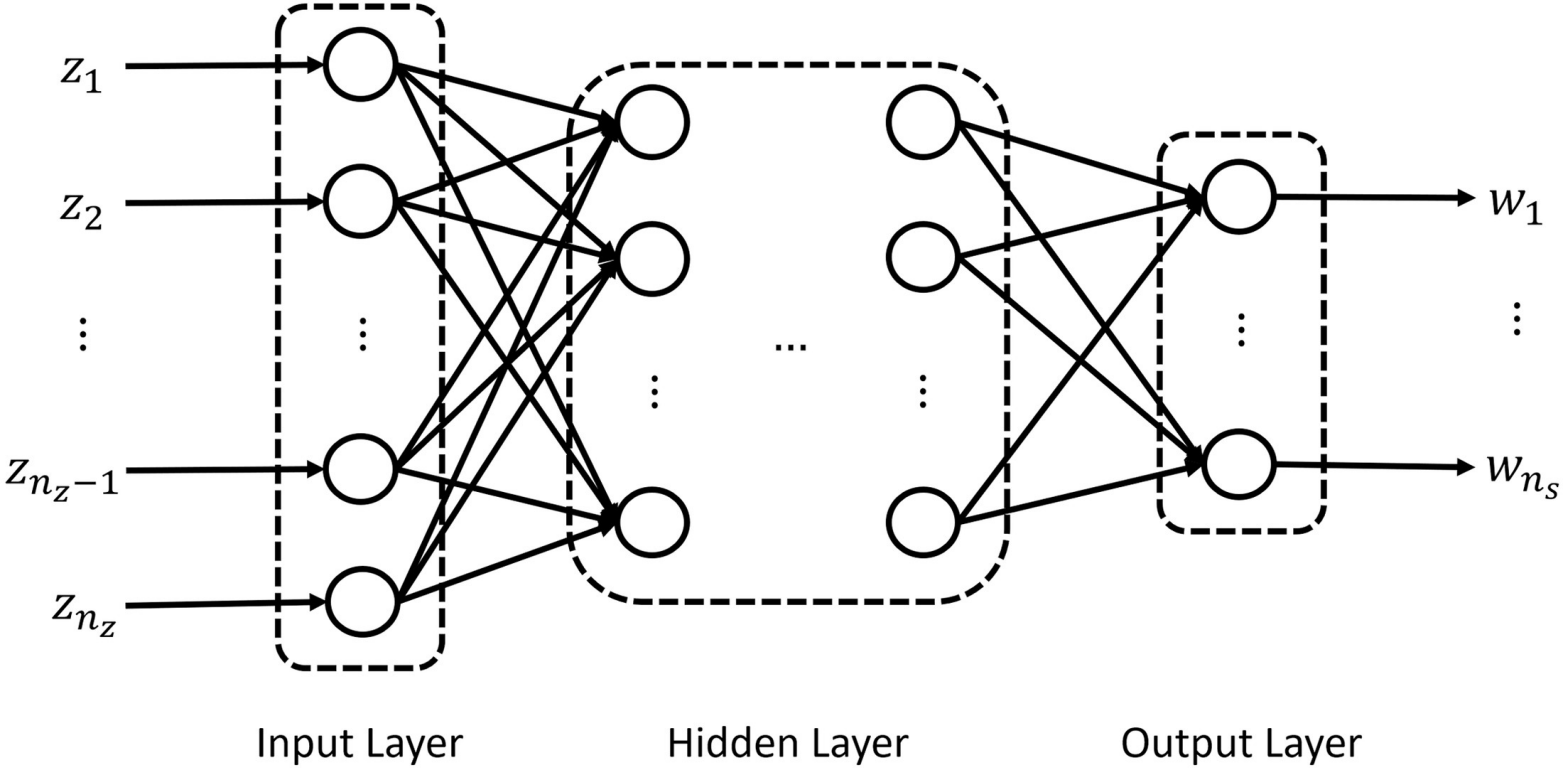
Schematic illustration of an ANN model.

An ANN consists of an input layer, multiple hidden layers, and an output layer. Specifically, each layer contains multiple neurons, and each neuron in each layer is fully connected to all the neurons in the next layer (Fig 2). In each hidden layer, the following hyperbolic tangent sigmoid transfer function is used:

$$\begin{array}{c} o_{i}^{\left(k\right)}=\frac{2}{1+e^{-2{\hat{u}}_{i}^{\left(k\right)}}}-1,\;{\hat{u}}_{i}^{\left(k\right)}=\sum_{j=1}^{N_{n}^{\left(k-1\right)}}(\alpha_{ij}^{\left(k\right)}\cdot o_{j}^{\left(k-1\right)}+b_{j}^{\left(k\right)}),\;\forall i=1,\ldots ,N_{n}^{\left(k\right)},\;k=1,\ldots ,N_{h} \end{array}$$ (12)

where $o_{i}^{\left(k\right)}$ is the output from the *i*^th^ neuron in the *k*^th^ hidden layer, ${\hat{u}}_{i}^{\left(k\right)}$ is the weighted sum of inputs given to the *i*^th^ neuron, $N_{n}^{\left(k\right)}$ is the number of neurons in the *k*^th^ hidden layer, $\alpha_{ij}^{\left(k\right)}$ is the weightage for the input $z_{j}^{\left(k\right)}$ to the *i*^th^ neuron, $b_{j}^{\left(k\right)}$ is the bias term given to the *i*^th^ neuron, and *N*_*h*_ is the number of hidden layers in an ANN. It should be noted that *o*^(*k*−1)^ for the first hidden layer is the inputs to an ANN.

On the other hand, the ANN outputs are computed as follows:

$$\begin{array}{c} {\hat{w}}_{c,l}=\sum_{i=1}^{Nn^{\left(N_{h}\right)}}\beta_{li}o_{i}^{\left(N_{h}\right)}+c_{l},\;l=1,\ldots ,n_{x} \end{array}$$ (13)

where ${\hat{w}}_{c,l}$ is the *l*^th^ element of ${\hat{w}}_{c}$, ${\hat{w}}_{c}$ is the predicted ***w***_*c*_ from the ANN, *β*_*li*_ is the weighthage given to $o_{i}^{\left(N_{h}\right)}$ for *w*_*l*_, and *c*_*l*_ is the bias term of *w*_*l*_.

#### Model selection and training

The goal of the ANN training is to estimate hyperparameters of an ANN model, which include *α*, *b*, *β*, and *c* in Eqs 12 and 13 from available datasets. Here, the datasets include ANN input and output datasets, where the ANN inputs refer to *t* and ***x***(*t*) from simulating the original first-principle model (Eq 1) while the ANN outputs refer to ***w*** estimated from solving Eq 5. All the ANN training sessions in this study are performed in the MATLAB Neural Network Toolbox with the Levenberg-Marquardt algorithm.

Since the structure of an ANN (i.e., the numbers of hidden layers and neurons in each hidden layer) is unknown beforehand, a number of ANN models with different structures are trained and compared to find the best one through evaluating their average corrected Akaike information criterion (AIC_c_) [57, 58]. For a model with *p* number of parameters, AIC_c_ can be calculated as follows:

$$\begin{array}{c} {\text{AIC}}_{c}=n\;\text{ln}\left(SSE/n\right)+n\frac{n+p}{n-p-2} \end{array}$$ (14)

where *n* is the number of data points in the dataset, *SSE* is the sum of squared errors between the observations and ANN predictions, and *p* is the number of the ANN hyperparameters.

To find an optimal structure, datasets are randomly partitioned into the training, testing, and validation sets with a 70:15:15 ratio 100 times, and ANN models with different structures are trained 100 times to compute their average AIC_c_ [59, 60]. Then, the ANN structure with the minimum AIC_c_ is selected as the optimal one.

Once an ANN is developed, the final mathematical form of a hybrid model can be described as follows:

$$\begin{array}{c} \begin{array}{cc} \dot{\tilde{x}}\left(t\right) & =f(\tilde{x},\theta ,u_{s};t)+H{\hat{w}}_{c}\left(t\right)\\ \tilde{y}\left(t\right) & =g(\tilde{x},u_{s};t)\\ {\hat{w}}_{c}\left(t\right) & =H(x\left(u_{s};t\right),t;h) \end{array} \end{array}$$ (15)

where ${\hat{w}}_{c}$ is the predicted value of ***w***_*c*_ from the developed ANN, H is the ANN developed as above, and ***h*** is a vector containing the ANN’s hyperparameters. It should be noted that the ANN inputs are *t* and ***x***, which are the model states simulated from the original first-principle model (Eq 1).

## Results

In this section, two case studies are presented to demonstrate how the proposed methodology can be implemented to develop a hybrid model of an intracellular signaling pathway.

### Case study 1: TNF*α* signaling pathway

**Fig 3:**
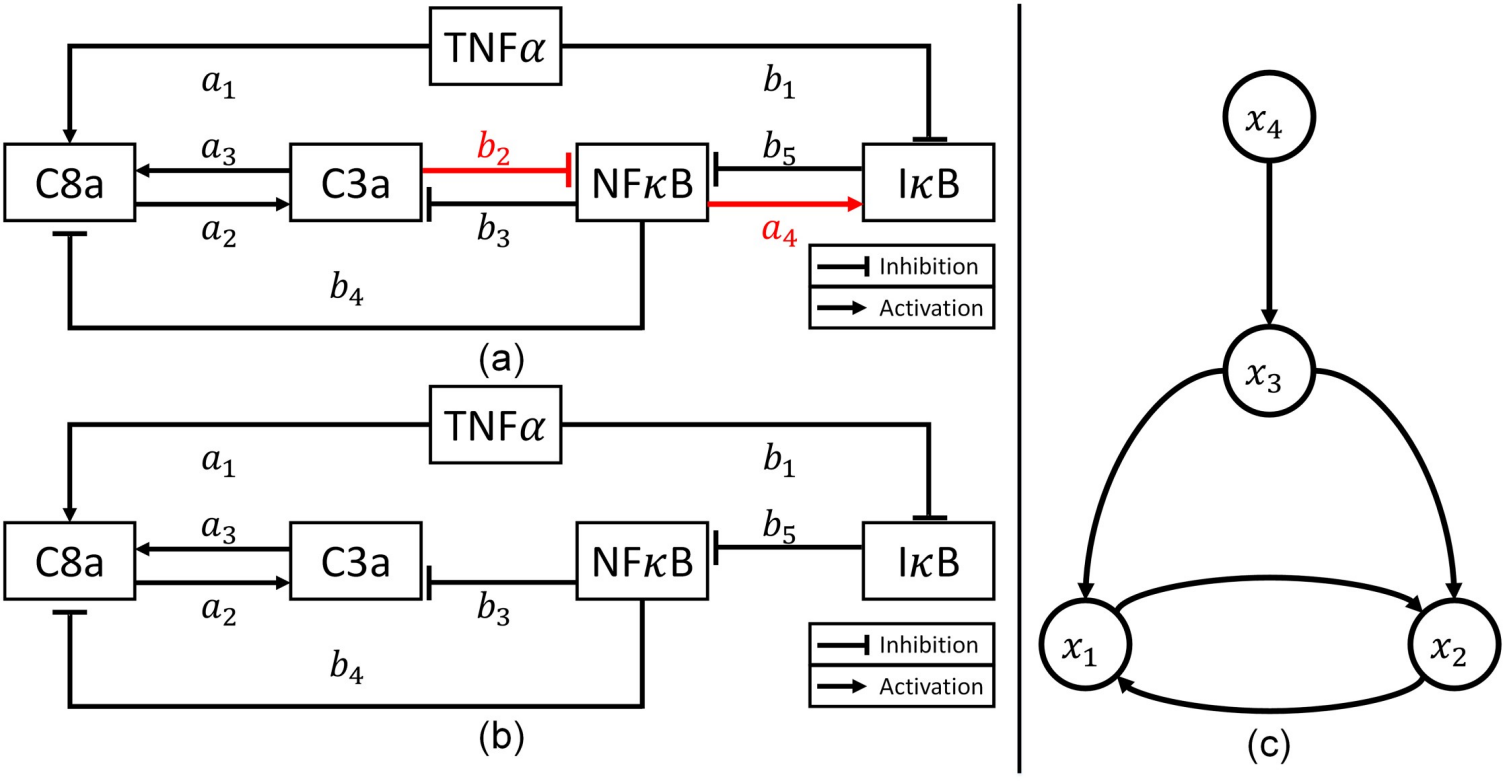
Schematic diagrams of the simplified NF*κ*B signaling pathway models. (a) The correct model that is used for generating *in silico* experimental measurements (adopted from Chaves et al. [61]). (b) The incorrect model that is used as the first-principle model for constructing a hybrid model. (c) A graph representation of the available (incorrect) first-principle model (Eq 19).

In this case study, the proposed scheme is first used to construct a hybrid model to describe a tumor necrosis factor-*α* (TNF*α*) signaling pathway, which is illustrated in Fig 3a. Specifically, this system describes how TNF*α*, which is an important inflammatory cytokine, can initiate apoptotic and nuclear factor-*κ*B (NF*κ*B) signaling pathways as well as crosstalks between these two pathways. In this system, the apoptotic signaling pathway is described by dynamics of caspase 3 (C3a) and caspase 8 (C8a), where C3a is a protein, whose high activity leads to apoptosis, and TNF*α*-activated C8a increases the C3a activity. On the other hand, TNF*α* activates NF*κ*B protein by suppressing inhibitor of NF*κ*B (I*κ*B), which inhibits the NF*κ*B activity. Since the NF*κ*B activation in turn increases the I*κ*B activity, the NF*κ*B activity will naturally decay over time. Furthermore, the NF*κ*B activation suppresses the C3a and C8a activities and thus promotes cellular survival, while the increase in the apoptotic signaling pathway lowers the NF*κ*B activity. More details on this system can be found in [61, 62].

#### *In silico* measurements

The TNF*α* signaling pathway represented in Fig 3a can be described by the following mathematical model [61, 62]:

$$\begin{array}{c} \begin{array}{cc} {\dot{x}}_{1} & =-x_{1}+\frac{1}{2}(inh_{4}\left(x_{3}\right)\cdot act_{1}\left(u\right)+act_{3}\left(x_{2}\right))\\ {\dot{x}}_{2} & =-x_{2}+act_{2}\left(x_{1}\right)\cdot inh_{3}\left(x_{3}\right)\\ {\dot{x}}_{3} & =-x_{3}+inh_{2}\left(x_{2}\right)\cdot inh_{5}\left(x_{4}\right)\\ {\dot{x}}_{4} & =-x_{4}+\frac{1}{2}(inh_{1}\left(u\right)+act_{4}\left(x_{3}\right)) \end{array} \end{array}$$ (16)

where *x*_*i*_, *i* = 1, …, 4, represents the relative activities of C8a, C3a, NF*κ*B, and I*κ*B, respectively, and *u* represents the TNF*α*. Also, the functions, *inh*_*i*_ and *act*_*i*_, in the model are rational functions given by:

$$\begin{array}{c} \begin{array}{cc} inh_{i}\left(x_{j}\right) & =\frac{x_{j}^{2}}{a_{i}^{2}+x_{j}^{2}}\\ act_{i}\left(x_{j}\right) & =\frac{b_{i}^{2}}{b_{i}^{2}+x_{j}^{2}} \end{array} \end{array}$$ (17)

where *a*_*i*_, *i* = 1, …, 5, and *b*_*i*_, *i* = 1, …, 4, are the model parameters whose values are shown in Table 1. The initial concentrations are [*x*_1_(0) *x*_2_(0) *x*_3_(0) *x*_4_(0)] = [0, 0, 0.29, 0.63].

**Table 1:** Nominal parameter values of the correct TNF*α* signaling model shown in Fig 3a.

Parameter	Value	Parameter	Value
*a*_1_	0.6	*b*_1_	0.4
*a*_2_	0.2	*b*_2_	0.7
*a*_3_	0.2	*b*_3_	0.3
*a*_4_	0.5	*b*_4_	0.5
		*b*_5_	0.4

In this case study, the model (Eq 16) is considered as the true system, and it is used to generate *in silico* experimental measurements. Specifically, the NF*κ*B activity (i.e., *x*_3_) is measured every hour from 0 to 14 hours under three different input conditions (i.e., *u* = 0.5, 1, 2). It should be noted that the value of *u* is fixed while generating the *in silico* measurements. Also, experimental noise is introduced as follows:

$$\begin{array}{c} y\left(t\right)=\mu_{\times }x_{3}\left(t\right)+\mu_{+} \end{array}$$ (18)

where *y*(*t*) is the measurement at time *t*, and *μ*_×_ is the multiplicative experimental noise term that is randomly sampled from a log-normal distributions (i.e., $\ln\mu_{\times }∼N\left(0,0.01\right)$), and *μ*_+_ is the additive noise term that is randomly sampled from a normal distribution N(0,0.01). This particular formulation is used since it is a realistic representation for the noise in measurements. Particularly, multiplicative noise (i.e., *μ*_×_) is often observed for non-negative data, and it is suggested to be one of main sources of variability in the biological data [63–66].

#### Available first-principle model

On the other hand, we assume that the system is only partially understood, and Fig 3b represents the current understanding of the system, which is described by the following ODE model:

$$\begin{array}{c} \begin{array}{cc} {\dot{\bar{x}}}_{1} & =-{\bar{x}}_{1}+\frac{1}{2}(inh_{4}\left({\bar{x}}_{3}\right)\cdot act_{1}\left(u\right)+act_{3}\left({\bar{x}}_{2}\right))\\ {\dot{\bar{x}}}_{2} & =-{\bar{x}}_{2}+act_{2}\left({\bar{x}}_{1}\right)\cdot inh_{3}\left({\bar{x}}_{3}\right)\\ {\dot{\bar{x}}}_{3} & =-{\bar{x}}_{3}+inh_{5}\left({\bar{x}}_{4}\right)\\ {\dot{\bar{x}}}_{4} & =-{\bar{x}}_{4}+\frac{1}{2}inh_{1}\left(u\right) \end{array} \end{array}$$ (19)

**Fig 4:**
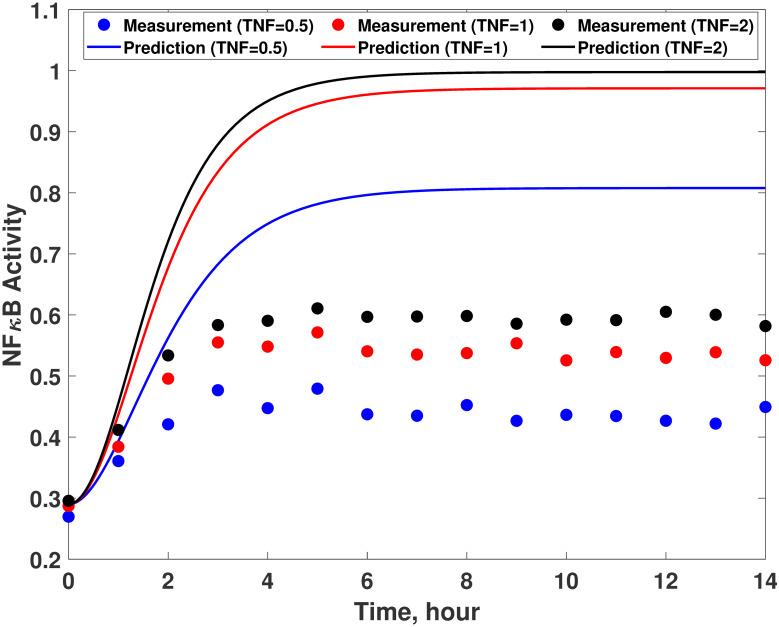
System-model mismatches in the TNF*α* signaling pathway model. Comparison between experimental measurements (empty circles) and model predictions (solid lines) with 0.5 (blue), 1 (red), and 2 (black) of TNF*α*. The measurements are generated by simulating Eq 16 while the model predictions are obtained from Eq 19.

Compared with the accurate system dynamics described by Eq 16, the imperfect first-principle model (Eq 19) misses two mechanisms in the true system: the C3a-induced suppression of NF*κ*B activity and NF*κ*B-induced I*κ*B activation. Due to such system-model mismatches, there is a considerable degree of discrepancy between measured and predicted dynamics as shown in Fig 4. Therefore, the proposed methodology is implemented to construct a hybrid model that can compensate for the model-system mismatches.

#### Hybrid model development

The first step of the proposed methodology is to determine a set of states whose trajectories need to be corrected by the addition of ***w***_*c*_. Since there is only one output (i.e., *x*_3_), the number of states to be corrected by correction terms is one as described earlier. Then, a graphical approach is implemented to determine which state should be corrected by *w*_*c*_. Fig 3c is a graphical representation of the first-principle model (Eq 19) to visualize the interconnections among the states. Since the number of the correction term to be added is determined to be one, the correction term can be added to one of *x*_1_, *x*_2_, and *x*_4_ as shown in S1 Fig. In Fig 3, it is clear that there is only one directed edge pointing to the output (*x*_3_), which stems from *x*_4_. Consequently, the only feasible configuration for the correction term in this system is the first one in S1 Fig, where the correction term is placed to adjust the dynamics of *x*_4_ and eventually the system output. It should be noted that the correction term is added to the differential equation of *x*_4_ only.

**Fig 5:**
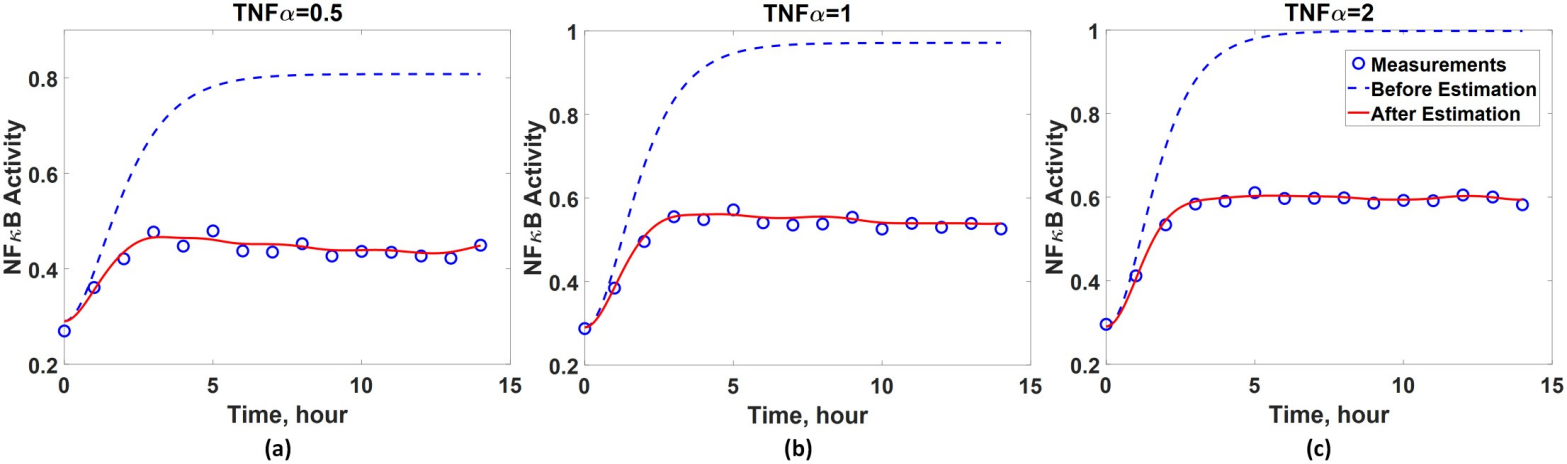
Validation of the accuracy of inferred *w*_*c*_. Comparison between predicted (red solid line) and measured (blue empty circle) NF*κ*B dynamics when the activity of TNF*α* equals to (a) 0.5, (b) 1, and (c) 2. Blue dash lines represent the model predictions without the correction terms (*w*_*c*_).

Next, the regularized least-squares problem is solved to estimate the values of *w*_*c*_ at the time instants when the measurements are taken under three different input conditions. As the *α* value in Eq 5 for this system is unknown, its optimal value is found by the five-fold cross-validation. Table 2 shows the average normalized mean squared errors (MSE) between model predictions and the measurements for five different *α* values, and the optimal *α* value is determined to be one. Hence, the *w*_*c*_ estimation results corresponding to *α* = 1 are used for the subsequent analysis and ANN development. Before constructing an ANN, the accuracy of the estimated values of *w*_*c*_ is assessed by comparing the experimental measurements and the dynamics predicted by the available (incorrect) first-principle model (Eq 19) coupled with the estimated *w*_*c*_. Fig 5 shows that the discrepancy between the predicted dynamics and the experimental measurements is diminished and thus validates the *w*_*c*_ estimation results.

**Table 2:** Comparison of the normalized MSE at different *α* values for the first case study.

*α*	normalized MSE
0.001	0.777
0.01	0.150
0.1	0.072
1	5.75 × 10^−3^
10	9.77 × 10^−3^

As the last step of the hybrid model construction, an ANN is developed to compute the *w*_*c*_ value. Here, inputs to the ANN are selected to be the values of states and input as well as the current time (i.e., [*x*_1_ *x*_2_ *x*_3_ *x*_4_ *u* *t*]), and the ANN output is the value of *w*_*c*_.

**Fig 6:**
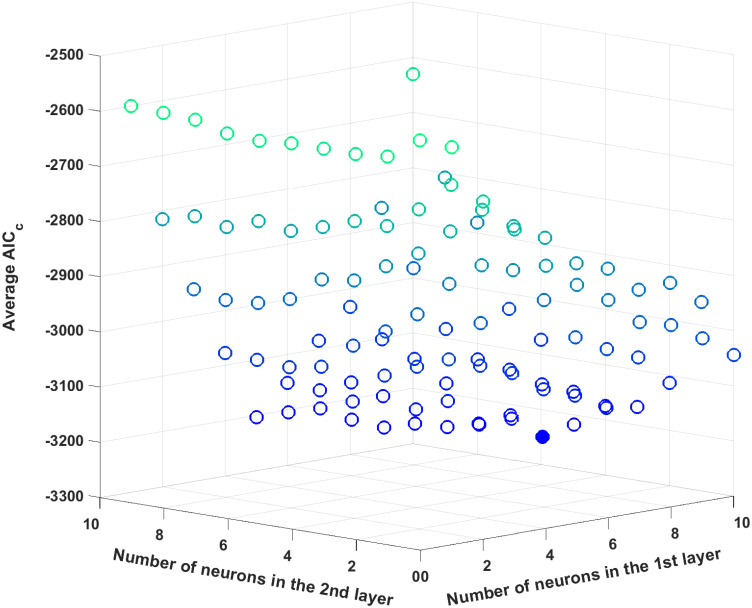
The average AIC_c_ values for different ANN structures for the first case study. The filled circle represents the minimum average AIC_c_ value.

The optimal number of hidden layers as well as the number of neurons in each hidden layer is to be optimized. As outlined earlier in the methodology, the AIC_c_ criterion is used to determine the ANN structure. To reduce the combinatorial complexity, the number of neurons in each hidden layer and the number of hidden layers are limited to ten and two, respectively. Then, each ANN is trained 100 times to compute the average AIC_c_ value with 100 different initial conditions for its hyperparameters, and the optimal ANN structure is determined by finding an ANN structure which results in the minimum average AIC_c_ value. Fig 6 plots the average AIC_c_ values for all possible ANN structures, and the AIC_c_ value reaches its minimum with eight and four neurons in the first and second hidden layers, respectively; hence, this particular structure is used for the subsequent analysis.

**Fig 7:**
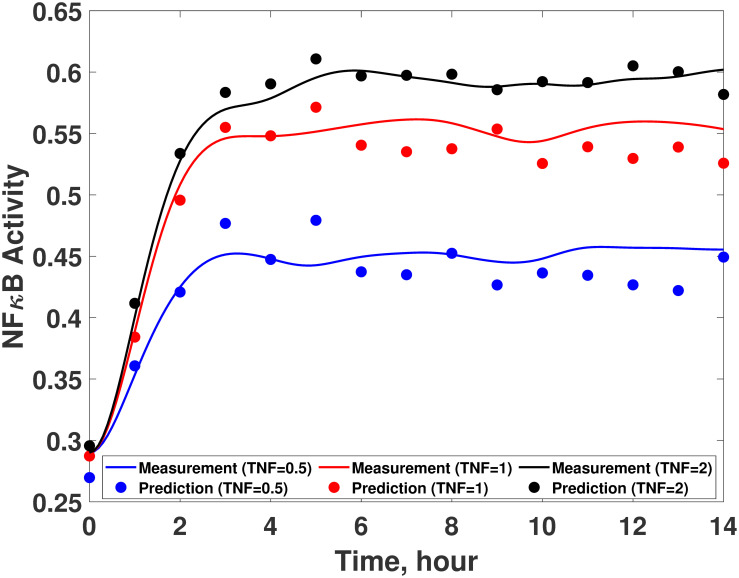
Validation of the developed hybrid model for the first case study. Comparison between experimental measurements (empty circles) and hybrid model predictions (solid lines) with 0.5 (blue), 1 (red), and 2 (black) of TNF*α*.

Among the 100 different ANNs with the optimal structure that have been trained in the previous step, the best ANN is chosen based on its *R*^2^ statistic value. Specifically, an ANN with the highest *R*^2^ value is chosen. As shown in Table 3, the *R*^2^ statistics of the best ANN are sufficiently high to ensure its accuracy. Then, this ANN is coupled with the available (incorrect) first-principle model (Eq 19) to finalize the hybrid model development. In order to validate the prediction accuracy of this hybrid model, it is simulated under three input conditions and compared with the experimental measurements. As shown in Fig 7, the developed hybrid model can describe the true system dynamics fairly accurately under all the conditions, and the MSE of the model prediction is reduced to 0.00027 from 0.12 which is the MSE value of the original first-principle model (Eq 19). This result shows that the hybrid model constructed by the proposed methodology can accurately describe the system dynamics even when there is limited knowledge on the underlying system (i.e., only a model with model-system mismatches is available from the literature).

**Table 3:** The *R*^2^ statistic values of the best ANN.

Training dataset	Validation dataset	Test dataset	Overall dataset
0.997	0.994	0.987	0.994

#### Prediction capability of the hybrid model

The hybrid model is analyzed further in this subsection to assess whether the developed hybrid model possess the desired features of a hybrid model: intrepretability and generalized prediction capability.

**Fig 8:**
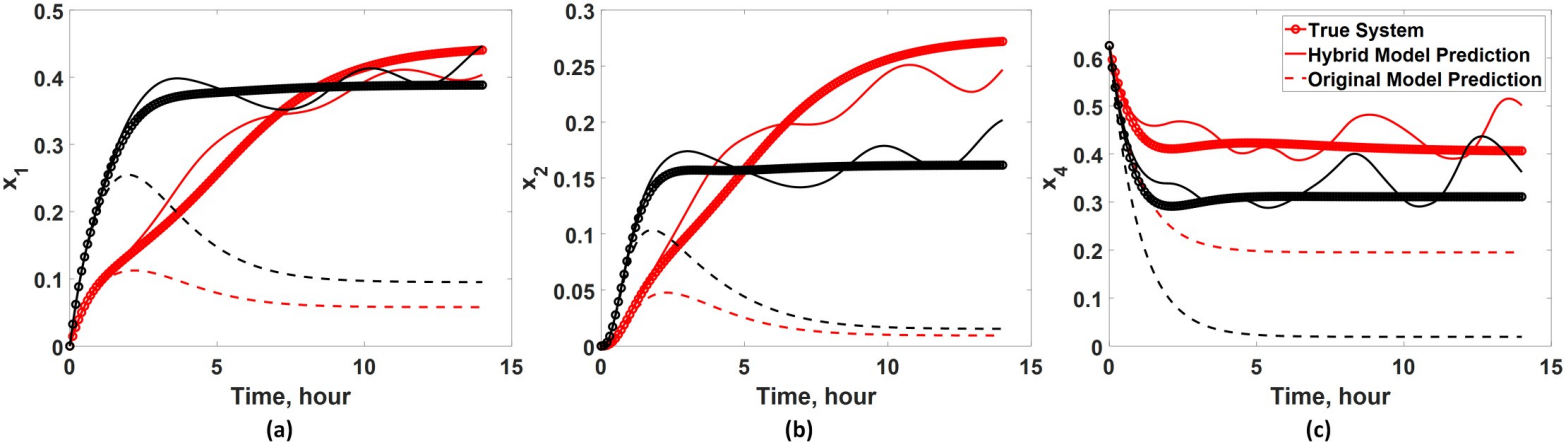
Validation of the interpretability of the hybrid model. The developed hybrid model is used to infer the dynamics of unmeasured states (i.e., (a) *x*_1_, (b) *x*_2_, and (c) *x*_4_) under two input conditions: *u* = 0.5 (red lines) and *u* = 2 (black lines). The hybrid model predictions are compared with the true system dynamics (Eq 16) and the available (incorrect) first-principle model (Eq 19).

First, the intrepretability of the developed hybrid model is tested by simulating and examining the temporal profiles of unmeasured states. Specifically, the dynamics of *x*_1_, *x*_2_, and *x*_4_ predicted by the developed hybrid model are compared with those of the true system (Eq 16) to assess whether the hybrid model can be used in predicting unmeasured states. As shown in Fig 8, the predictions from the developed hybrid model agree well with the true system dynamics (Eq 16) and show significant improvement in the prediction accuracy compared with the available (incorrect) first principle model (Eq 19). Particularly, it is remarkable to note that the hybrid model can predict the dynamics of *x*_1_ and *x*_2_ quite well even though *w*_*c*_ is added only to *x*_4_ for correcting its dynamics. Such improvement is possible since the hybrid model has incorporated the existing (known) interactions among the model states via the first-principle model (Eq 19).

**Fig 9:**
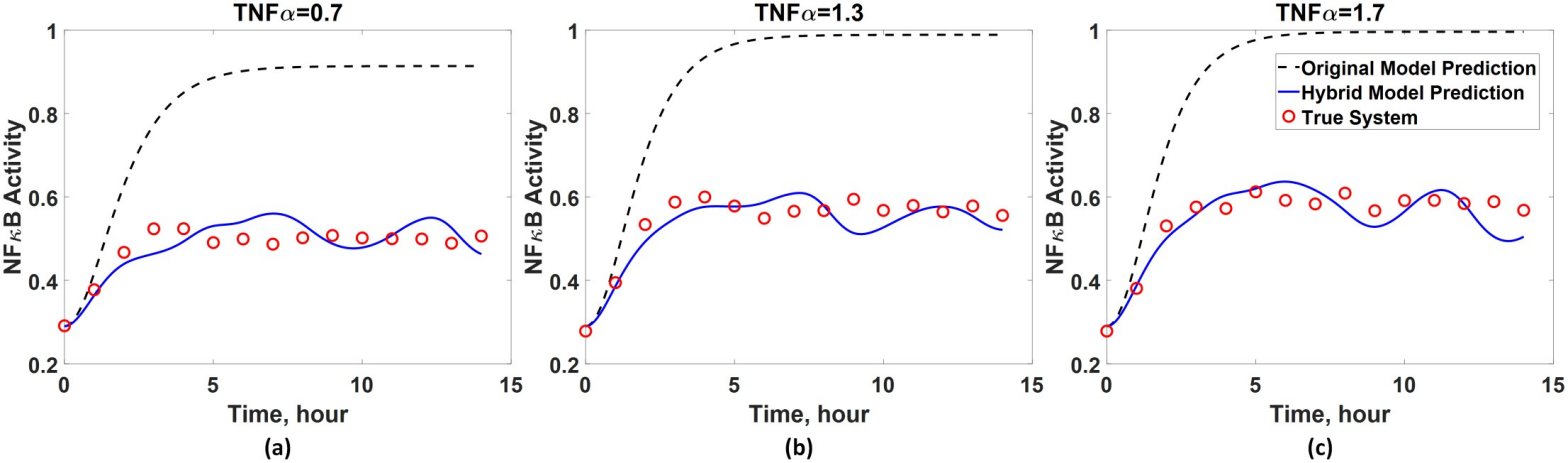
Generalized prediction capability of the developed hybrid model for the first case study. The prediction accuracy of the hybrid model is assessed by comparing with true system dynamics when the activity of TNF*α* equals to (a) 0.7 and (b) 1.7. The dynamics predicted by the original first-principle model (Eq 19) are also plotted for the comparison.

Additionally, it is of great interest to know whether the developed hybrid model has a generalized prediction capability. To this end, the hybrid model is used to predict the dynamics under three different input conditions (i.e., *u* = 0.7, 1.3, 1.7), and the model predictions are compared with the true system dynamics obtained from Eq 16. Here, these three particular input conditions are chosen since they lie within the input range used for training the ANN, but they are not identical to the inputs. As shown in Fig 9, the first-principle model as expected fails to capture the NF*κ*B dynamics accurately. However, the hybrid model is capable of predicting the dynamics of *x*_4_ fairly accurately. These results show that the hybrid model possesses the generalized prediction capability due to the incorporation of an ANN. In summary, this case study highlights advantages of using a hybrid model over a purely data-driven model or first-principle one since a hybrid model can essentially improve the overall model prediction accuracy while maintaining the model interpretability.

#### Robustness of hybrid model

Additionally, since it is imperative to understand how the presence of measurement noise might impact the performance of the proposed methodology, different levels of noise are introduced by varying the distributions for *μ*_×_ and *μ*_+_ in Eq 18 to examine the impact of the measurement noise. Specifically, a noiseless dataset (i.e., *μ*_×_ = 1 and *μ*_+_ = 0) and that with a higher noise level ($\ln\mu_{\times }∼N\left(0,0.05\right)$ and $\mu_{+}∼N\left(0,0.05\right)$) are generated, and their corresponding hybrid models are constructed. The additional datasets as well as the original dataset are plotted in S2 Fig to illustrate their difference in the noise level. With these additional datasets, the proposed methodology is implemented to develop the corresponding hybrid models, and S3 Fig illustrates the model accuracy of the hybrid models developed based on the noiseless and more noisy datasets, respectively. MSE of the developed hybrid models based on the noiseless and more noisy measurements is 0.00015 and 0.0048, respectively, while that of the hybrid model in Fig 7 is 0.00027. Overall, regardless of the noise level in the measurements, the developed hybrid models had significantly improved prediction capability as their predicted dynamics show reasonable agreements with the measurements. To further assess the impacts of the measurement noise, the interpretability of the hybrid models is assessed by using them to predict the dynamics of unobserved states as shown in S4 and S5 Figs. Overall, all the hybrid models were able to predict the dynamics of the unobserved states with reasonable accuracy.

However, it should be noted that both in the MSE and intrepretability analysis, the oscillations in the predicted dynamics become more noticeable with a higher level of noise. Since the oscillations are not present in the true dynamics, this comparison shows that the noise level may negatively influence the accuracy of a hybrid model as well as its interpretability. Also, even the hybrid model developed from the noiseless measurements produces the dynamics with nontrivial oscillations, which indicates that the ANN might have been overfitted. In order to mitigate such a problem, the future work in this direction could incorporate the following ideas to improve the hybrid model performance. First, a de-noise technique can be implemented prior to the hybrid model development so that the noise in the measurements will become less influential. In the literature, various methods such as finite difference with polynomial spline [67], spectral transformation [68], sparse Bayesian regression [69], and neural networks [70] have been proposed. Second, alternative ANN training mechanisms can be implemented to improve the performance of an ANN. So far, an ANN is trained with the Levenberg-Marquardt algorithm through Matlab Neural Network Toolbox. Since the presence of the oscillations suggests the potential overfitting issues in the ANN training, Bayesian regularization training method [71] may be implemented to mitigate this issue.

In a systems biology study, the parameter estimation is usually performed to quantitatively calibrate an uncertain model based on available measurements. In the literature, numerous studies have proposed a wide variety of methods to efficiently estimate parameter values, and these methods have been implemented to successfully model various biological systems [2, 72, 73]. However, if there is significant model-system mismatch, the parameter estimation alone may not be enough for calibrating a model. In such a circumstance, the proposed hybrid modeling approach can be implemented. As an example, the parameter estimation is performed for this system to see whether it is enough to train the model (Eq 19). Here, the parameter estimation is preceded by global sensitivity analysis to determine a subset of parameters that are identifiable from available measurements (see [74, 75] for details). The result of the sensitivity analysis is shown in Table 4, where *ϕ*_*D*_ represents the sensitivity index of a parameter set. Based on the magnitude of the sensitivity index, it is determined that *b*_1_ and *b*_5_ are identifiable. Then, a least-squares problem is solved to estimate their values by minimizing the difference between the model predictions and the measurements (i.e., *in silico* measurements simulated from Eq 16). The estimated values of *b*_1_ and *b*_5_ are 1 and 0.30, respectively, and the accuracy of the calibrated model is assessed by comparing the model predictions with the measurements as show in S6 Fig. It is clear that the parameter estimation alone is not enough to overcome the underlying structural mismatch between the model and the true system for this signaling pathway. In summary, the parameter estimation alone may not be enough for quantitatively calibrating the model if there is a significant degree of model-system mismatch. And, the proposed hybrid modeling approach will be a valuable option to construct an accurate and physically meaningful model.

**Table 4:** The sensitivity analysis results.

Parameter subsets	*ϕ*_*D*_
*b*_1_	17.25
*b*_5_	15.18
*b*_1_, *b*_5_	137.4

### Case study 2: NF*κ*B signaling pathway dynamics induced by LPS and BFA

While the previous case study demonstrates how the proposed methodology can be applicable to a relatively simple system and the *in silico* measurements, the second case study will examine how the proposed scheme can be used to develop a hybrid model for a much more complex system with real *in vitro* measurements. Specifically, a hybrid model is developed to describe the lipopolysaccharide (LPS)-induced NF*κ*B signaling pathway in the presence of brefeldin A (BFA).

As briefly described in the previous case study, the NF*κ*B signaling pathway is involved in the apoptotic signaling pathway, but it is also involved in a number of different cellular processes such as inflammation and differentiation [76, 77]. Our previous study [75] aimed to model how the NF*κ*B signaling pathway can be activated by LPS, an endotoxin derived from gram-negative bacteria [78], in the presence of BFA. One of the major products of the signaling pathway is TNF*α* protein, which is a pro-inflammatory cytokine and propagates inflammatory signals to adjacent cells [79, 80]. While the LPS-induced NF*κ*B signaling pathway is relatively well studied and has been previously modeled in the literature [81, 82], the impact of BFA on the overall signaling pathway is less known. It is suggested that BFA activates the NF-*κ*B signaling pathway by activating another signaling pathway called the endoplasmic reticulum (ER)-stress pathway, which will subsequently initiate the NF-*κ*B [75, 83]. Since the ER-stress pathway itself and how it activates the NF*κ*B signaling pathway have not been fully elucidated yet [84, 85], it is very difficult to formulate an accurate mechanistic model to describe the overall signaling dynamics. In our previous study, we introduced time-varying functions to represent the effects of the BFA addition on the NF*κ*B signaling pathway. By the introduction of new mechanisms and subsequent parameter estimation, the model accuracy was improved significantly, but there was still noticeable discrepancy between the model prediction and the measurements. Also, developing the time-varying components was also a nontrivial task, which involved further literature review and experimentation. In this study, the proposed hybrid modeling approach is to develop a hybrid model to infer the effects of the BFA addition on the dynamics of the LPS-induced NF*κ*B signaling pathway from the measurements.

**Fig 10:**
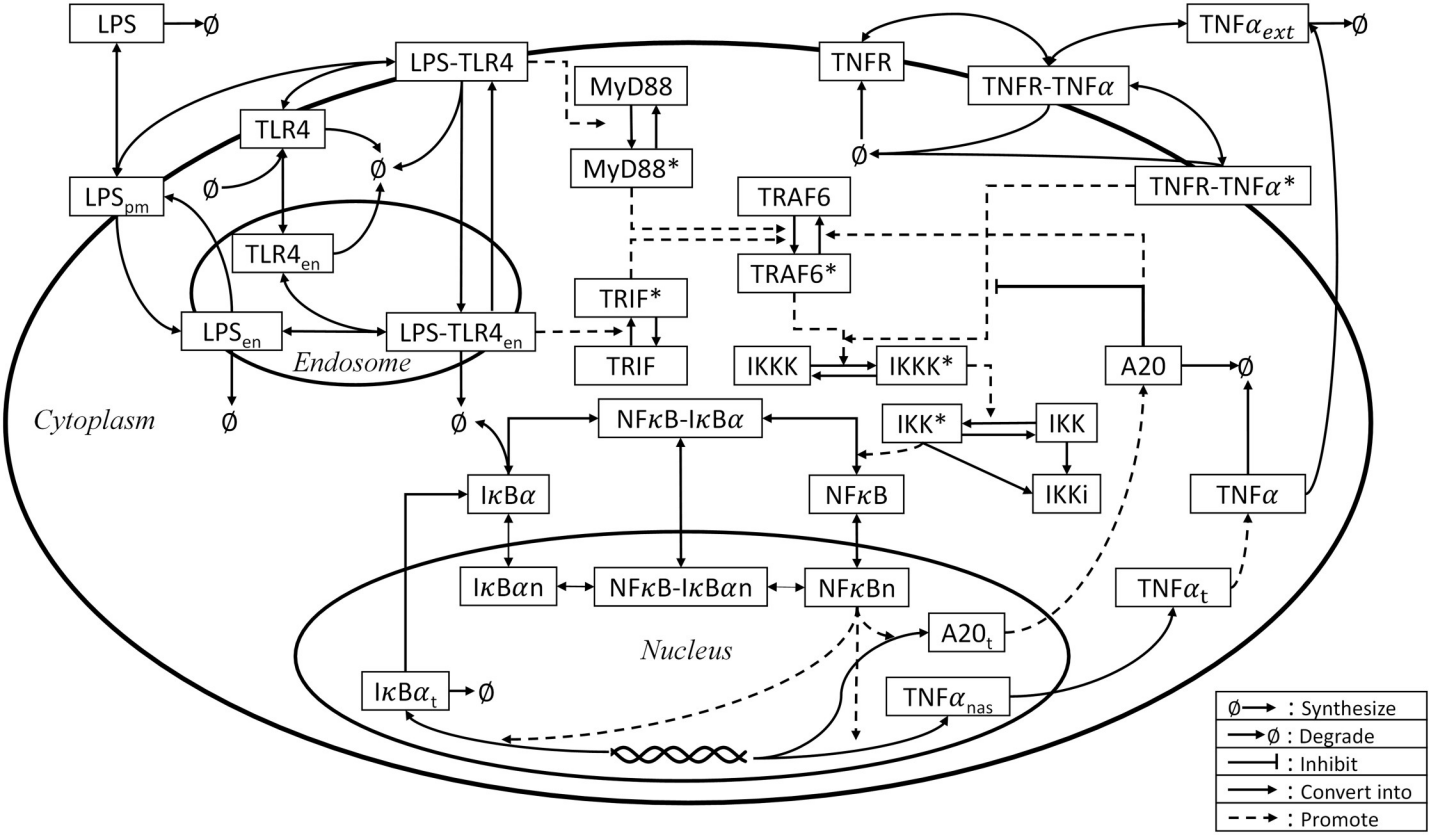
A schematic illustration of the LPS-induced NF*κ*B signaling pathway.

The first-principle model that will serve as the basis of the hybrid model to be developed is adopted from our previous study [75]. This model describes how LPS can induce the NF*κ*B activation and TNF*α* synthesis (Fig 10) by a system of nonlinear ODEs. This model consists of 49 states and 149 parameters, and a detailed description on the model can be found in [75].

In our previous study [75], RAW 264.7 murine macrophages were stimulated by LPS in the presence of BFA, and the dynamics of the NF*κ*B signaling pathway were measured by flow cytometry. Specifically, we measured fold changes of TNF*α* and I*κ*B*α*, which are two important proteins in the overall NF*κ*B signaling pathway, under three different LPS concentrations in the presence of BFA. As a result, the model outputs are defined as the fold change of the two proteins with respect to their initial conditions:

$$\begin{array}{c} \begin{array}{cc} y_{1}\left(t\right) & =\frac{\text{TNF}\alpha (t)}{\text{TNF}\alpha (t_{0})}\\ y_{2}\left(t\right) & =\frac{\text{I}\kappa \text{B}\alpha (t)}{\text{I}\kappa \text{B}\alpha (t_{0})} \end{array} \end{array}$$ (20)

where *y*_1_(*t*) and *y*_2_(*t*) are the fold changes of TNF*α* and I*κ*B*α* concentrations, respectively, at time *t*. It should be noted that the measurements were taken at eight time instants, that is, ***t***_*s*_ = [0, 10, 20, 30, 60, 120, 240, 360] minutes after addition of LPS.

#### *w*_*c*_ estimation

As outlined in the previous section, the first step in developing a hybrid model is to determine the number of correction terms needed as well as to which states these correction terms should be added. As the number of outputs for the system of interest is two (i.e., TNF*α* and I*κ*B*α*), the dimension of ***x***_*c*_ is also two.

Second, all possible permutations of choosing two from 49 model states are enumerated, and their corresponding digraphs are constructed to compute their corresponding relative order matrices. Based on the constructed relative order matrices, the minimum value of $\sum_{i=1}^{n_{y}}r_{i}$ is found to be two, and Table 5 lists all the configurations whose $\sum_{i=1}^{n_{y}}r_{i}$ values are equal to two. It should be note that *r*_1*j*_ and *r*_2*j*_ compute the relative order with respect to I*κ*B*α* and TNF*α* measurements, respectively, in Table 5. Based on the result presented in Table 5, *x*_5_ and *x*_37_ are chosen as the best candidates to add ***w***_*c*_ since this pair has the lowest ∑_*i*_ ∑_*j*≠*i*_ *r*_*i*_/*r*_*ij*_ value. Here, *x*_5_ and *x*_37_ represent concentrations of I*κ*B*α* and TNF*α* transcripts, respectively. Therefore, adding correction terms to these states is a reasonable choice since the predicted dynamics of a protein will become more accurate with more understanding of its transcript dynamics.

**Table 5:** Selection of optimal *w*_*c*_ locations by minimizing relative-order based criteria.

*x*_*s*1_	*x*_*s*2_	*r*_11_	*r*_12_	*r*_21_	*r*_22_	$\sum_{i=1}^{n_{y}}r_{i}$	∑_*i*_ ∑_*j*≠*i*_ *r*_*i*_/*r*_*ij*_
5	37	1	6	6	1	2	0.33
1	37	1	6	5	1	2	0.37
3	37	1	6	5	1	2	0.37
34	37	1	6	5	1	2	0.37
2	37	1	6	4	1	2	0.417
4	37	1	6	4	1	2	0.417
5	28	1	4	6	1	2	0.417
1	28	1	4	5	1	2	0.45
3	28	1	4	5	1	2	0.45
34	28	1	4	5	1	2	0.45
2	28	1	4	4	1	2	0.50
4	28	1	4	4	1	2	0.5

With ***x***_*c*_ = [*x*_1_ *x*_37_], the regularized least-squares problem (Eq 5) is solved to estimate ***W*** that contains the values of ***w***_*c*_ at eight time points for each LPS concentration. Since the value of the regularization parameter *α* in Eq 5 is unknown beforehand, its optimal value is determined by the five-fold cross-validation. Table 6 shows the values of normalized MSE with respect to different values of *α*. From this result, the optimal value of *α* is determined to be 0.001, and the estimated values of ***W*** obtained with *α* = 0.001 are considered to develop a hybrid model.

**Table 6:** Comparison of the normalized MSE at different *α* values for the second case study.

*α*	normalized MSE
1 × 10^−5^	0.0275
0.0001	0.0283
0.001	0.0181
0.01	0.0187
0.1	0.0235
1	0.0310
10	0.0390

**Fig 11:**
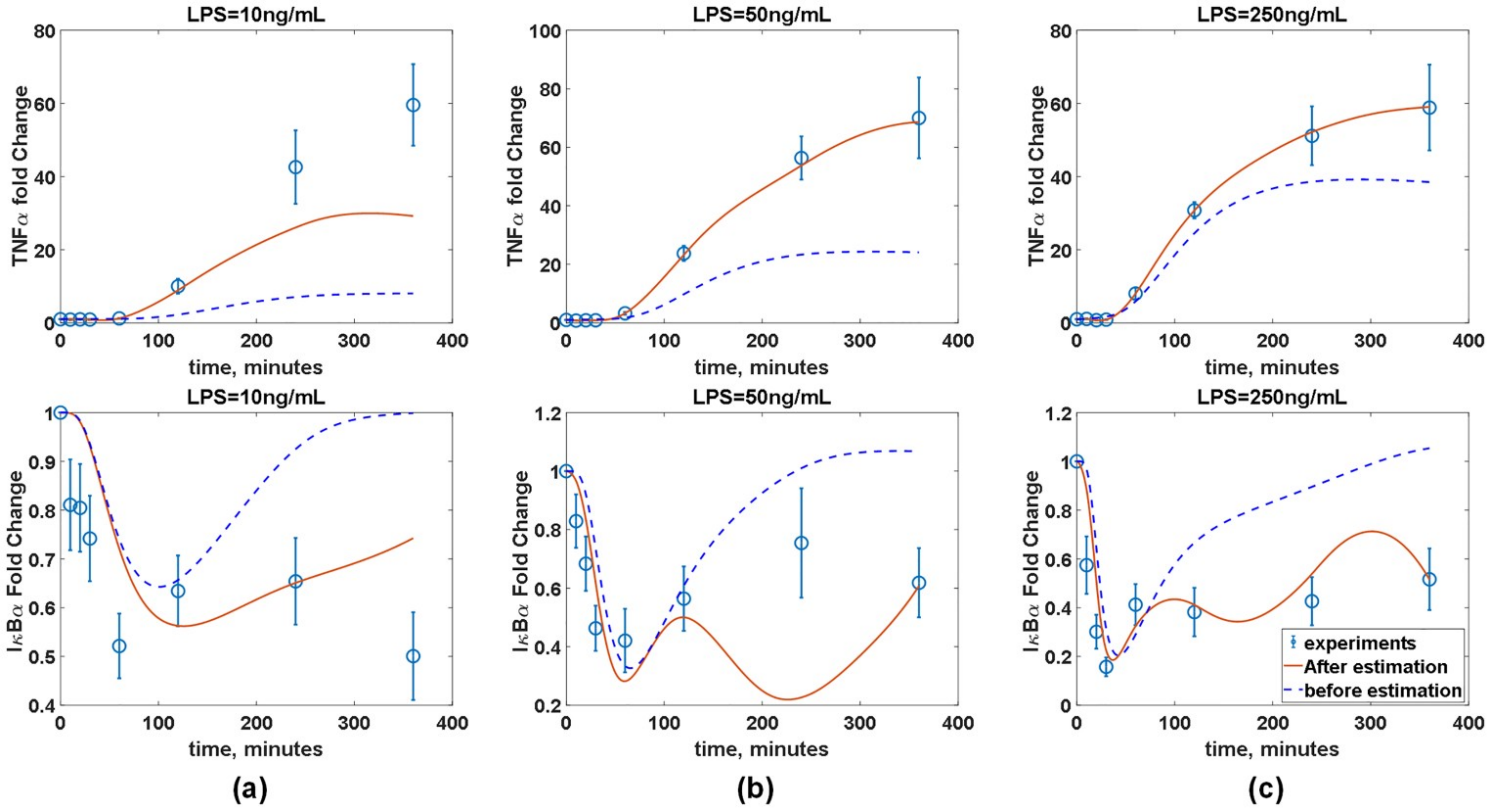
The model prediction accuracy is improved by coupling the available (imperfect) first-principle model with the estimated *w*_*c*_. Red solid lines and blue empty circles represent simulated and measured TNF*α* dynamics and I*κ*B*α*, respectively, in the presence of BFA under three different LPS concentrations. Blue dash lines represent the model predictions without the correction terms (***w***_*c*_).

Before constructing an ANN model, the accuracy of the inferred ***W*** is assessed by comparing the experimental measurements and predictions from the imperfect model coupled with the inferred ***W***. It should be noted that ***w***_*c*_ is linearly interpolated to obtain its values at the time instants when the measurements are not taken. Fig 11 compares the predicted and measured TNF*α* and I*κ*B*α* dynamics under three different LPS concentrations. Additionally, the predictions of the model coupled with the inferred ***W*** are compared with those of the imperfect model without ***W***. Fig 11 shows that the addition of ***W*** significantly improves the model accuracy across all three LPS concentrations. Specifically, the addition of ***W*** renders the model prediction to match with trends observed in the experiments, which show the sustained low concentration of I*κ*B*α*. At the same time, the addition of ***W*** helps the model predictions agree much better with the measured TNF*α* dynamics. Overall, these comparisons have demonstrated that the integration of ***W*** greatly improves the predictive capability of the available (imperfect) first-principle model.

**Fig 12:**
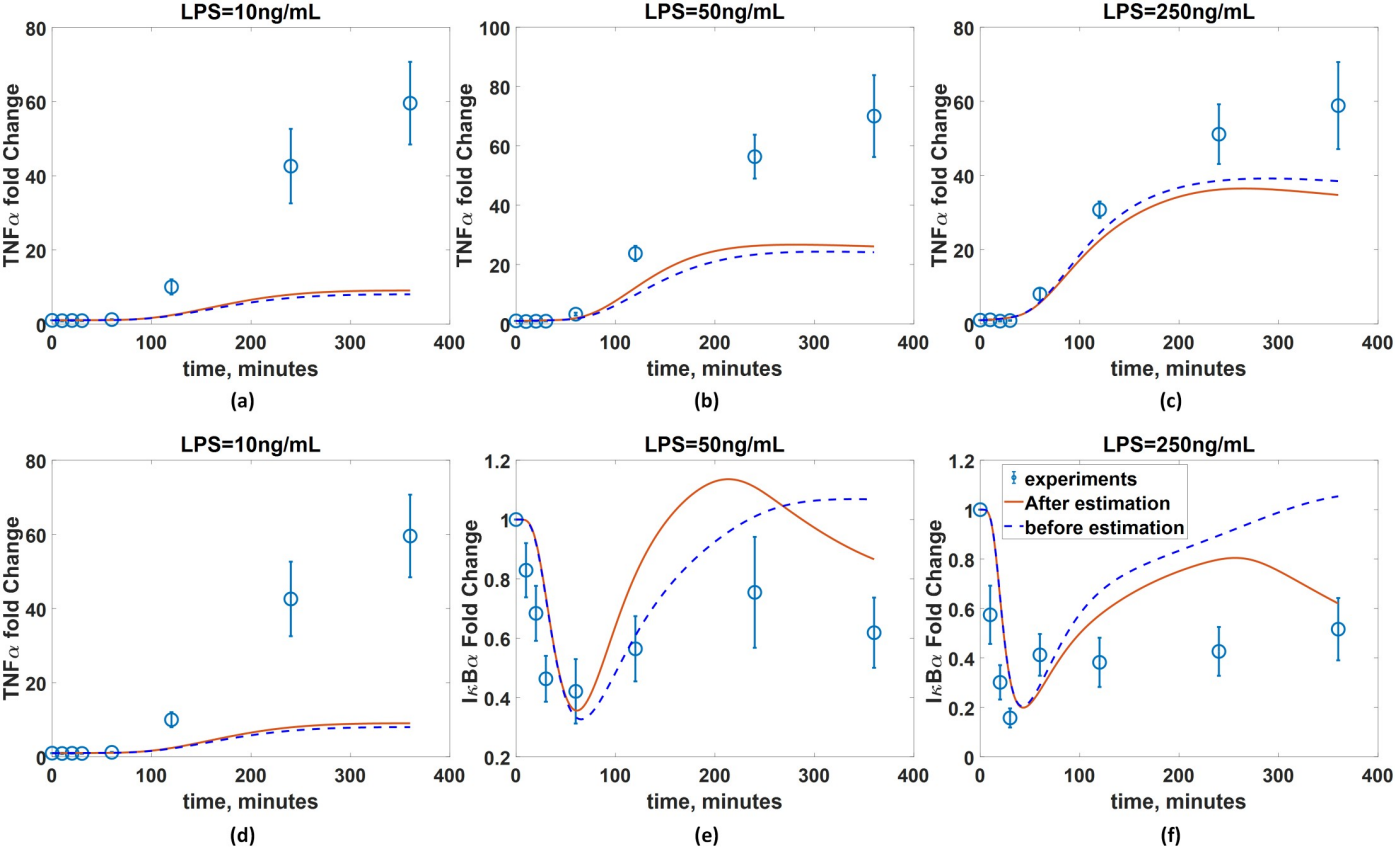
Importance of selecting an optimal *x*_*c*_. ***w***_*c*_ are added to ***x***_*c*_ = {2, 17} as explained in the test. Red solid lines and blue empty circles represent simulated and measured TNF*α* dynamics and I*κ*B*α*, respectively, in the presence of BFA under three LPS concentrations. Blue dash lines represent the model predictions without the correction terms (***w***_*c*_).

Additionally, in order to demonstrate the necessity of choosing an optimal ***x***_*c*_, a different ***x***_*c*_ is chosen, and their corresponding values of ***W*** are estimated. Specifically, ***x***_*c*_ = {2, 17} is selected, and their selection criterion values are $\sum_{i=1}^{n_{s}}=4$ and $\sum_{i}^{n_{s}}\sum_{j\neq i}r_{i}/r_{ij}=1.25$, which are much higher than those of the optimal choice of ***x***_*c*_. Then, the first-principle model is coupled with the estimated ***w***_*c*_, and its predictions are compared with the available measurements. Fig 12 illustrates that addition of ***w***_*c*_ does not significantly improve the accuracy of the model prediction, which highlights the validity of determining the optimal set of ***x***_*c*_ from Table 5 based on the relative-order criteria.

#### ANN development

With the inferred ***W***, an ANN is developed to generalize the relationship between the first-principle model and ***w***_*c*_ values. Specifically, inputs and output of the ANN are [***x***(*t*), *t*] and ***w***_*c*_(*t*), respectively.

**Fig 13:**
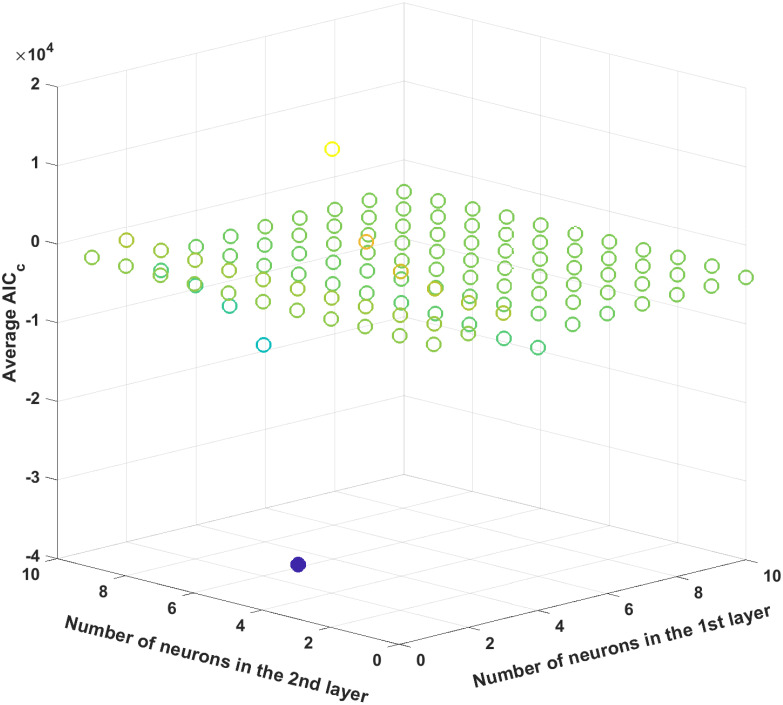
The average AIC_c_ values for different ANN structures. The filled circle represents the minimum average AIC_c_ value.

Next, the ANN structure is optimized by minimizing the average AIC_c_ values. Similar to the previous case study, we will limit the numbers of hidden layers and neurons in each hidden layer to two and ten, respectively, and each ANN structure is trained 100 times. Fig 13 plots the average AIC_c_ value for all possible ANN structures, and the one with three and six neurons in the first and second hidden layers, respectively, is shown to be optimal since it provides the minimal average AIC_c_ value. Then, among 100 different trained ANNs with this particular structure, the best ANN is selected by its *R*^2^ statistics. Table 7 shows the *R*^2^ values of the chosen ANN, which are all above 0.98 and thus demonstrate its prediction accuracy.

**Table 7:** The *R*^2^ statistic valued of the best ANN for the second case study.

Training dataset	Validation dataset	Test dataset	Overall dataset
0.998	0.999	0.986	0.997

**Fig 14:**
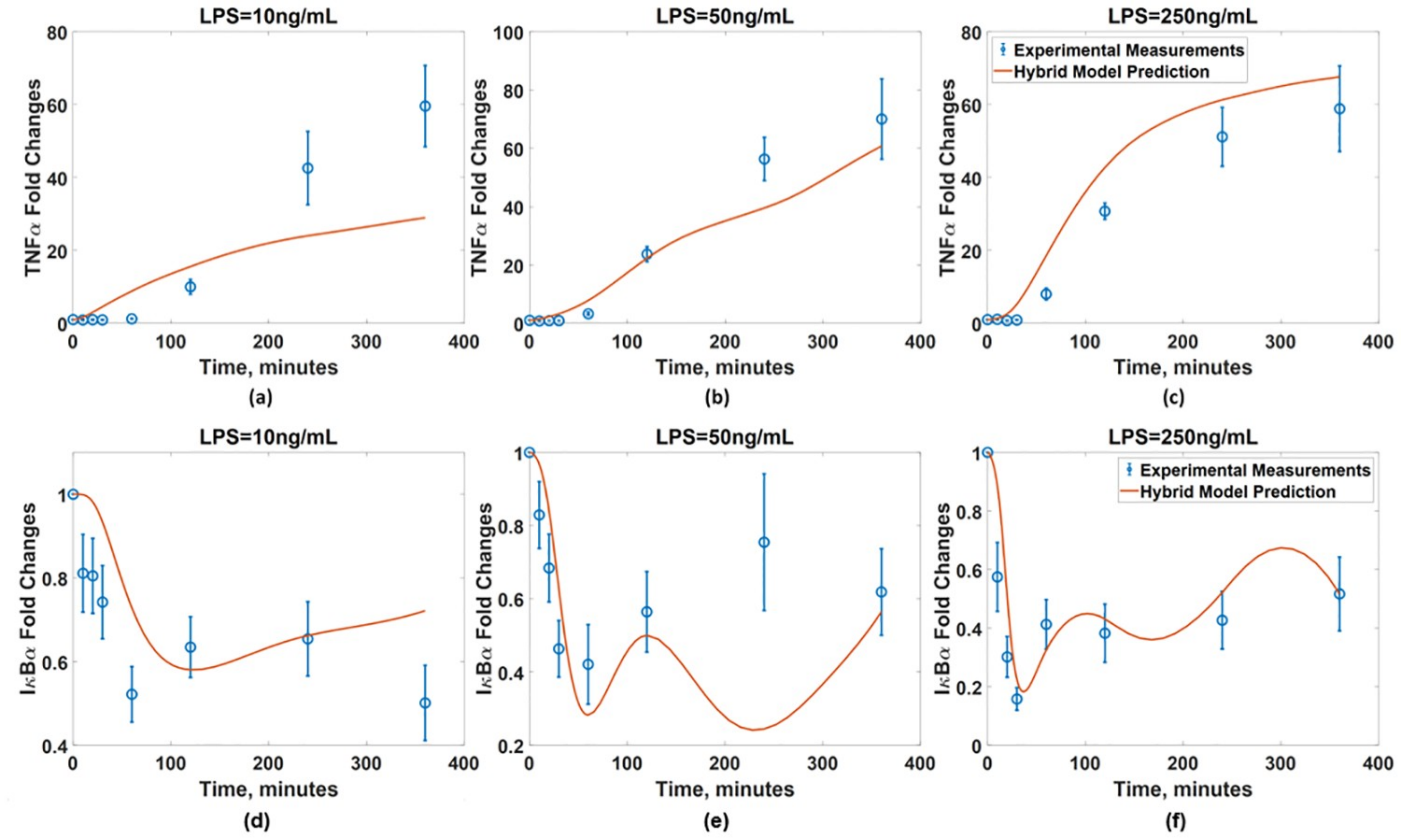
Validation of the developed hybrid model. The I*κ*B*α* dynamics predicted from the hybrid model with the developed ANN (red solid line) are compared with the measurements (blue empty circle) in the presence of BFA under the LPS concentrations of (a) 10ng/mL, (b) 50ng/mL, and (c) 250ng/mL. The TNF*α* dynamics predicted from the hybrid model with the developed ANN (red solid line) are compared with the measurements (blue empty circle) under the LPS concentrations of (d) 10ng/mL, (e) 50ng/mL, and (f) 250ng/mL. Blue dash lines represent the model predictions without the ANN.

The developed ANN is then coupled with the available (imperfect) first principle-model to finalize the hybrid model. Fig 14 compare the predicted dynamics from the resulted hybrid model with the experimental measurements. The normalized MSE values of the hybrid model are 1.95 and 6.3 × 10^−4^ with respect to the TNF*α* and I*κ*B measurements, respectively, while those of the original first-principle model are 12.5 and 7.0 × 10^−3^, respectively.

This shows that the hybrid model with the developed ANN has generalized the prediction capability of the first-principle model coupled with the experimentally inferred ***w***_*c*_; as a result, the developed hybrid model now can be utilized to predict the dynamics of the NF*κ*B signaling pathway in new conditions.

Although the developed hybrid model greatly improves the prediction accuracy, the model-system mismatch still remains. Specifically, under LPS = 10 ng/mL, the predicted dynamics of both TNF*α* and I*κ*B*α* do not perfectly agree with the measurements. Specifically, the hybrid model predicts a monotonic increase in the I*κ*B*α* dynamics and thus attenuation of TNF*α* synthesis, which indicates that the NF*κ*B activity gradually decays during this time period. On the other hand, the measurements show that the TNF*α* synthesis slows down beyond 240 minutes while the I*κ*B*α* level decreases after 240 minutes, which is not consistent with the model predictions. There is an additional mismatch in the I*κ*B*α* dynamics under LPS = 50 ng/mL. Specifically, the hybrid model predicts an oscillatory behavior with two troughs at 60 and 240 minutes and a peak at 120 minutes while the experimental measurements indicate a monotonic increase from 60 to 240 minutes without an intermediate peak. Overall, such remaining model-system mismatches demonstrate that the BFA addition has more pronounced effects on the overall signaling dynamics after 200 minutes. This has been documented in our previous work [75], where the dynamics of I*κ*B*α* in the presence of BFA deviate from those of I*κ*B*α* without BFA after 200 minutes. Increasing the dimension of ***w***_*c*_ may further improve the prediction accuracy due the increase in the degree of freedom. Alternatively, the first-principle model can be modified further by incorporating known mechanisms of the BFA-induced signaling pathways to improve the first-principle model before estimating the values of ***w***_*c*_. Specifically, it was suggested that the addition of BFA will elicit another signaling pathway called ER stress signaling pathway [75], which will suppress the translation of I*κ*B*α*. In the future, this mechanism can be incorporated into the first-principle model to improve the accuracy of the hybrid model.

## Discussion

In this work, we have presented a systematic way to construct a hybrid model that can accurately describe the dynamics of an intracellular signaling pathway even when we have partial understanding of the system. In order to simulate the dynamics of a signaling pathway of interest, prior understandings of the system are formulated into a system of nonlinear ODEs as its first-principle model. Since the first-principle model incorporates underlying mechanisms of the system, the model can be used to predict the system dynamics under new conditions and infer unmeasured model states’ dynamics once the model is properly calibrated by experiments [2–4, 86]. However, the development of such a first-principle model is nontrivial, and one of the largest bottlenecks in the model development process is lack of fundamental knowledge that may lead to the inaccurate formulation of a first-principle model.

Under such circumstances, data-driven modeling approaches such as proper orthogonal decomposition [15], subspace identification [17] and partial least squares regression [25] are commonly implemented to derive a dynamic model of a system whose corresponding first-principle model is too difficult to be formulated or is computationally too costly. Such models are advantageous to accurately derive empirical relationships between inputs and outputs. However, these models are difficult to be generalized and usually require a large amount of observations. To this end, this study proposes to use a hybrid model to improve prediction accuracy by combining the characteristics of both first-principle and data-driven modeling approaches. The available, albeit incomplete, knowledge of the system is used in a hybrid model, and the model-system mismatches are incorporated by inferring unknown components’ dynamics (i.e., ***W***) from experimental measurements [87]. In order to construct a hybrid model systematically, the presented work proposes a sequential two-step approach. First, ***x***_*c*_ and its dynamics are identified and estimated through the graphical approaches and solving an L2-regularized least-squares problem. Second, an ANN model is developed to correlate the available (imperfect) first-principle model with the values of ***w***_*c*_.

Through the proposed hybrid modeling approach, the developed hybrid model is able to posses prediction generalizability as a first-principle model does through the incorporation of the first-principle model coupled with an ANN. That is, a hybrid model can accurately predict the unmeasured states’ dynamics, and it can also be used to predict the system dynamics under a new operating condition as shown in two cases studies. At the same time, the hybrid model is likely to have more accurate predictions by inferring and incorporating the dynamics of components (i.e., ***w***_*c*_), which are missing in the first-principle model [29]. Such representations of the hidden components with an empirical function such as an ANN is particularly attractive when mechanistic understandings are completely lacking or only partially known, which may result in highly uncertain mechanistic equations with unreliable model predictions.

## Supporting information

S1 Fig Three possible locations of *w* for the first case study. (TIF)

S2 Fig Three different levels of noise introduced in *in silico* measurements in the first case study. (TIF)

S3 FigPrediction accuracy of two additional hybrid models in the first case study.These hybrid models are developed based on (a) the noiseless measurements and (b) the measurements with ln $\mu_{\times }∼N\left(0,0.05\right)$ and $\mu_{+}∼N\left(0,0.05\right)$, respectively.(TIF)

S4 Fig Further assessment of the hybrid model developed from the noiseless measurements. The developed hybrid model is used to predict the dynamics of unobserved states.
(TIF)

S5 FigFurther assessment of the hybrid model developed based on the measurements with more noise in the measurements (i.e., $\ln\mu_{\times }∼N\left(0,0.05\right)$ and $\mu_{+}∼N\left(0,0.05\right)$).The developed hybrid model is used to predict the dynamics of unobserved states.(TIF)

S6 Fig Results of the parameter estimation for the first case study without developing a hybrid model. (TIF)
