# Supplementary figures and images for: Development of a hybrid model for a partially known intracellular signaling pathway through correction term estimation and neural network modeling
Source: PLoS Comput Biol. 2020 Dec 14;16(12):e1008472. doi: 10.1371/journal.pcbi.1008472 (PMC7769624; doi:10.1371/journal.pcbi.1008472)

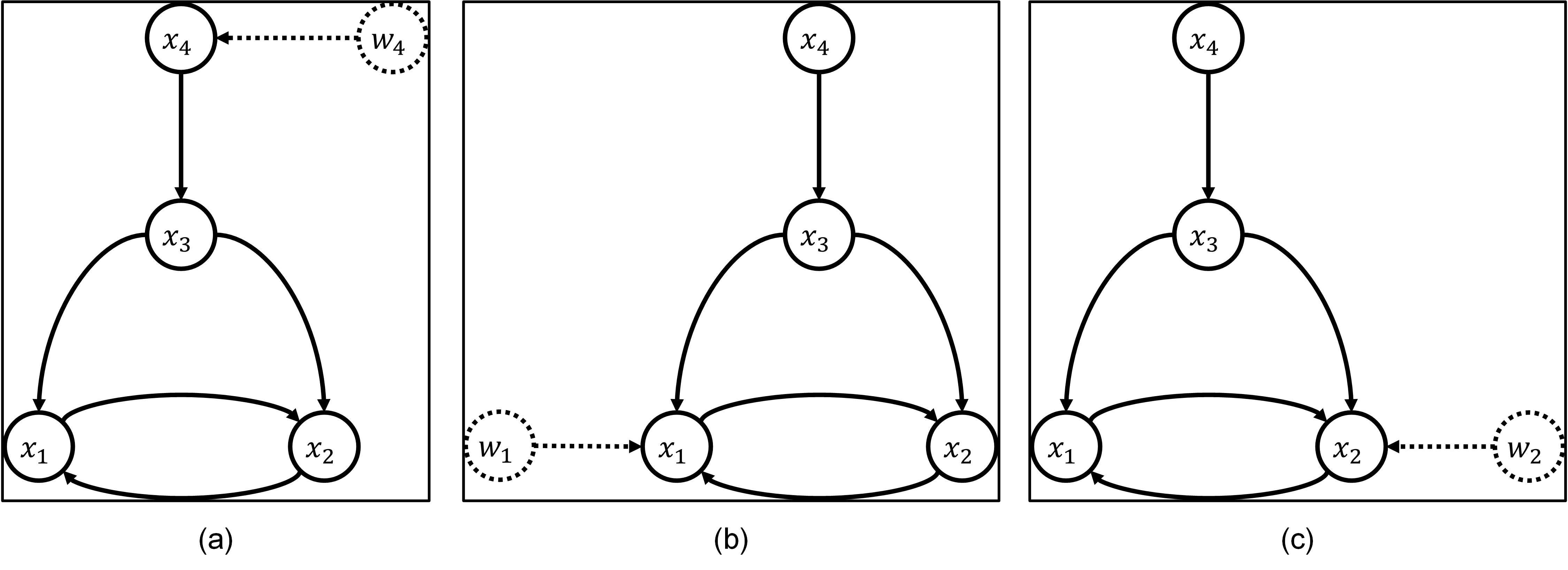

Supplement: S1 Fig — (TIF) [file pcbi.1008472.s001.tif]

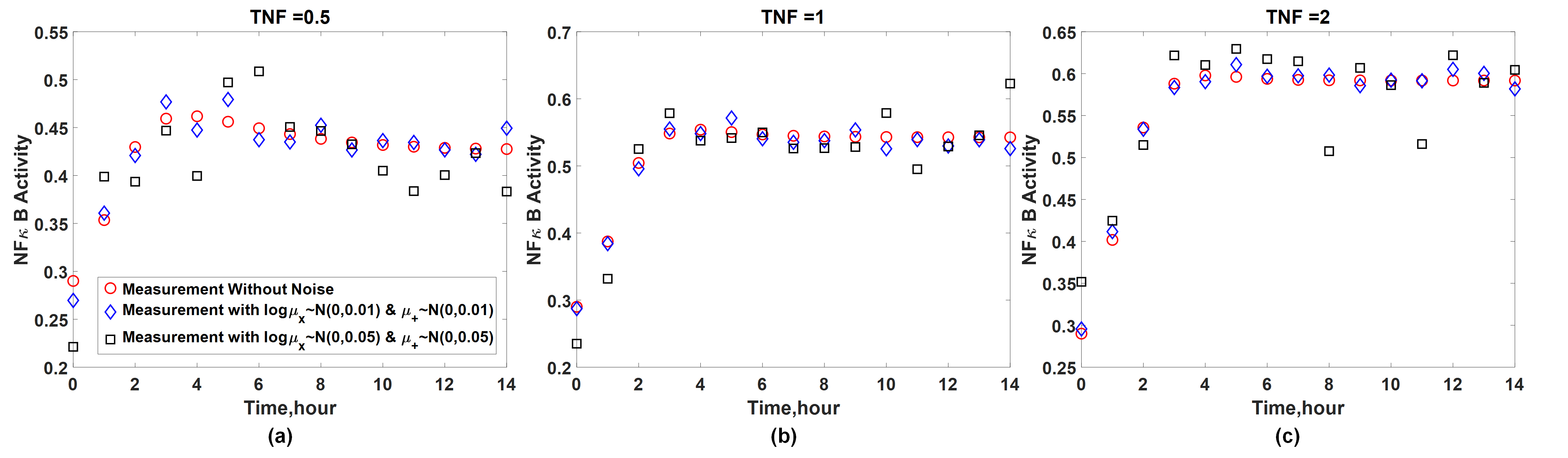

Supplement: S2 Fig — (TIF) [file pcbi.1008472.s002.tif]

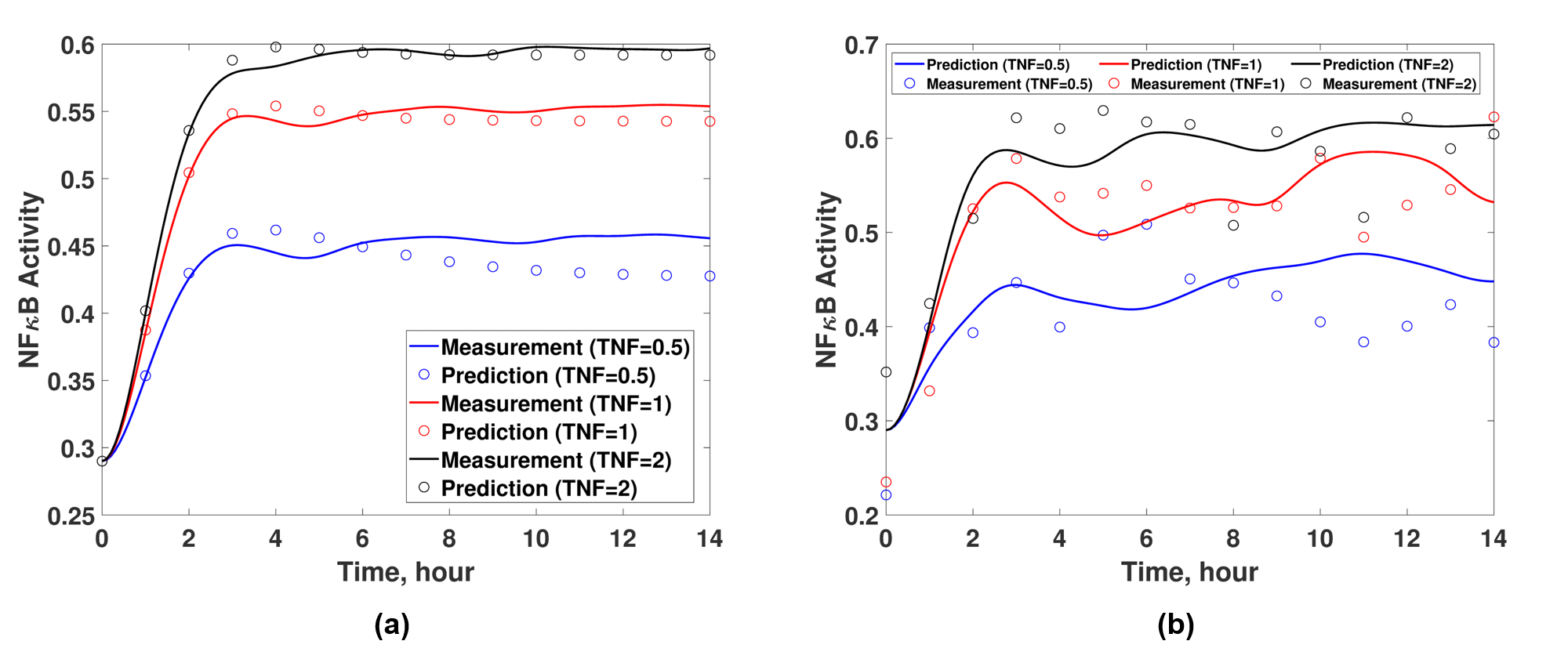

Supplement: S3 Fig — These hybrid models are developed based on (a) the noiseless measurements and (b) the measurements with ln μ×∼N(0,0.05) and μ+∼N(0,0.05), respectively. (TIF) [file pcbi.1008472.s003.tif]

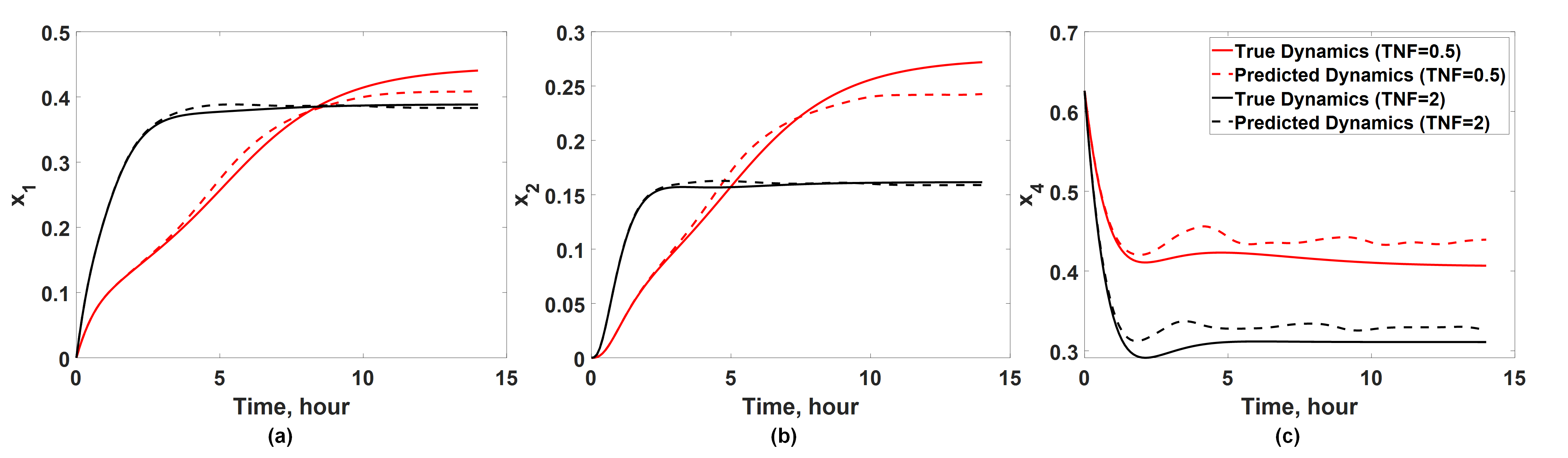

Supplement: S4 Fig — The developed hybrid model is used to predict the dynamics of unobserved states. (TIF) [file pcbi.1008472.s004.tif]

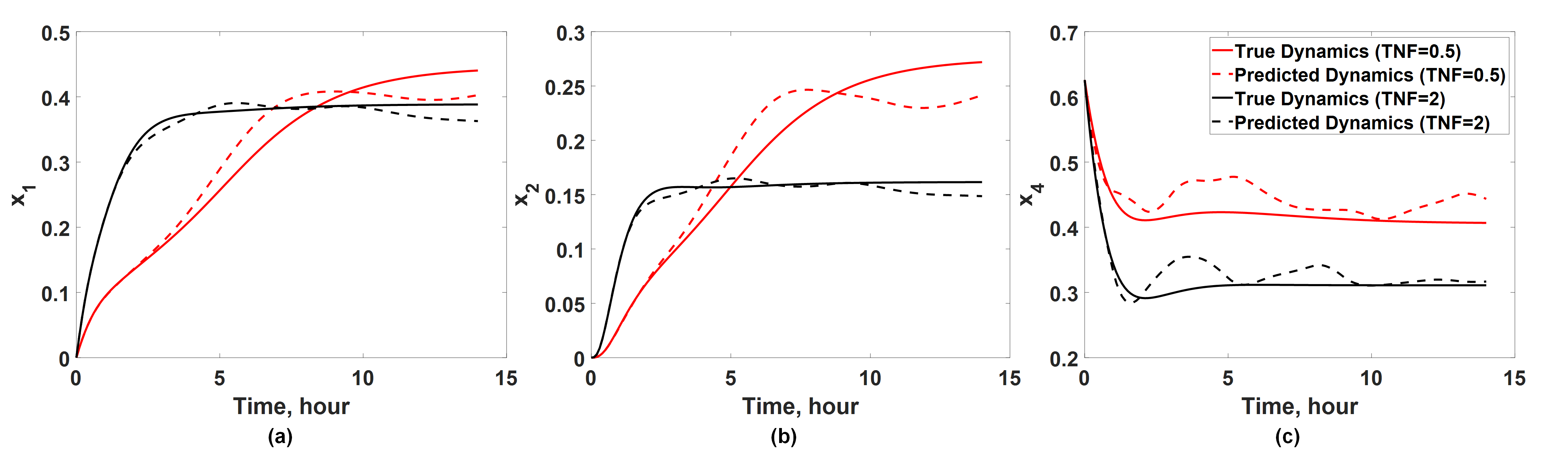

Supplement: S5 Fig — The developed hybrid model is used to predict the dynamics of unobserved states. (TIF) [file pcbi.1008472.s005.tif]

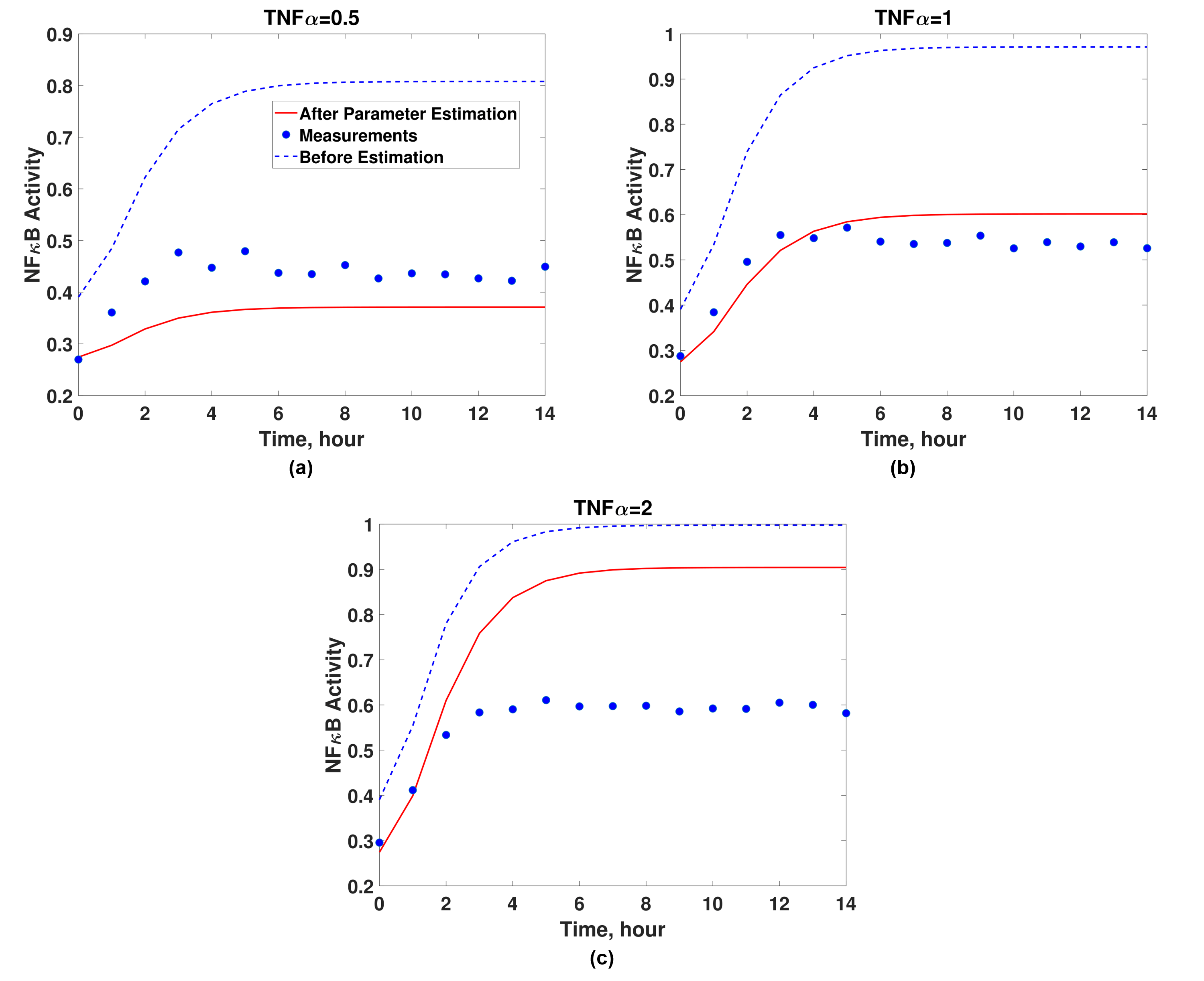

Supplement: S6 Fig — (TIF) [file pcbi.1008472.s006.tif]
